# Scoping Review of the Socioeconomic Value of Working Equids, and the Impact of Educational Interventions Aimed at Improving Their Welfare

**DOI:** 10.3390/ani16020165

**Published:** 2026-01-07

**Authors:** Amelia Cameron, Sarah L. Freeman, Isabella Wild, Jessica Burridge, Katie Burrell

**Affiliations:** 1 School of Veterinary Medicine and Science, University of Nottingham, Loughborough LE12 5RD, UK; sarah.freeman@nottingham.ac.uk (S.L.F.); katie.burrell1@nottingham.ac.uk (K.B.); 2 World Horse Welfare, Anne Colvin House, Snetterton, Norwich NR16 2LR, UK; wildiv@cardiff.ac.uk

**Keywords:** working equids, equine welfare, one welfare, one health, socioeconomics, educational interventions, human behaviour change, evidence reviews, low- and middle-income countries, sustainable development goals

## Abstract

Working equids support millions of people globally, especially in low-income, lower-middle-income, and upper-middle-income countries. However, they commonly suffer from poor welfare and are typically overlooked in policy and funding decisions. This scoping review aimed to collate evidence on two topics related to working equid use in low- and middle-income countries: their socioeconomic value to their owners and the impact of educational interventions for owners/handlers aiming to improve equid welfare. Original research published from 2014 onwards was eligible for inclusion. This scoping review followed the JBI methodology and PRISMA-ScR framework. One search strategy encompassing both topics was applied to five databases (CAB Abstracts, MEDLINE, Embase, Web of Science, and IBSS) on 24.04.24. Key characteristics and findings of eligible studies were charted. In total, 3514 sources were independently screened by two reviewers. In total, 61 socioeconomic value studies (47 journal articles, 2 reports, and 12 conference contributions) and 23 educational intervention studies (11 journal articles and 12 conference contributions) were included. Working equids supported their owners’ livelihoods in wide-ranging ways and contributed to the United Nations’ Sustainable Development Goals. Educational interventions employed varied approaches, and most reported success. Multilevel initiatives and those developed through participatory engagement may be more likely to directly improve equid welfare in the long term. These aspects should be prioritised during intervention development. The included studies used inconsistent terminology and were of variable quality. This review highlights the importance of including working equids within policy and funding strategies and provides recommendations to increase the discoverability, quality, and impact of working equid research.

## Simple Summary

Millions of people in low- and middle-income countries rely on work carried out using horses, donkeys, and mules to survive. This includes generating income, saving time and money, and reducing labour. However, these equids’ welfare is often poor, and they receive little consideration in policy and funding decisions. This scoping review examined two areas: the economic and social importance of working equids and the effectiveness of educational interventions and training programmes designed to improve equid welfare. Five databases were searched for studies published since 2014, and over 3500 sources were screened. In total, 84 studies met the criteria: 61 on socioeconomic value and 23 on educational interventions. The findings show that working equids perform wide-ranging roles, such as agriculture, domestic tasks, and transport of people and goods. They provide significant support to their owners’ livelihoods and contribute to achieving sustainable development goals. Educational interventions were generally successful in improving owner/user knowledge and behaviour and/or equid welfare. Approaches developed with input from target communities and that addressed the issue on multiple levels may be more effective. The terminology used and study quality varied. This review highlights the importance of considering working equids in policy and funding decisions and provides recommendations to improve future research.

## 1. Introduction

There are an estimated 120 million equids globally, with 87% of these residing in low- and middle-income countries (LMICs) [1]. The vast majority of equids in LMICs perform working roles [2,3], and they are often referred to as ‘working equids’. Although accurate data on the number of these animals is lacking [4], working equids in LMICs are often essential to the livelihoods of their owners. Working equids (horses/ponies, donkeys, and their hybrids (mules and hinnies)) can provide their owners with a route out of debt and extreme poverty [5,6], generating income through means such as transport, tourism, and agriculture [7,8,9,10]. They also contribute to domestic tasks, which typically fall to women, including transporting water and firewood [11]. Equid ownership can also have social benefits, such as providing transport to visit family, allowing the use of equids in cultural events, and improving social status and relationships when equids are loaned to others [11,12,13]. Women can also gain respect in their community by being seen to handle their equid [11]. Therefore, working equids can have a significant socioeconomic contribution to their owners and the wider communities in which they are kept. However, working equids have not been prioritised in policy-making and funding decisions from government bodies and funding agencies [14,15]. Grace et al., 2022 [9] posit that working equids are not sufficiently recognised as contributing to the United Nations’ Agenda 2030 Sustainable Development Goals (SDGs) [16] and outline the contributions they make to many of the SDGs. Socioeconomic data is required to fully understand and recognise working equids’ importance and contributions to their owners and communities. Research has been carried out to systematically map the available evidence in the context of the socioeconomic impact of working equid disease [12,17,18]. However, this systematic mapping has not been conducted for research into the wider socioeconomic impact of working equid ownership, which would provide a more comprehensive picture of the value of working equids in LMICs.

Working equids often have poor welfare, with common issues including lameness, poor body condition, wounds, colic, infectious disease, and parasites [7,12,19,20,21]. Many factors contribute to compromised welfare, such as a lack of access to veterinary services, and a lack of knowledge of equine husbandry and how to recognise and treat disease and injuries [22]. Long work hours, limited rest opportunities, overloading, unsuitable tack and equipment, and a lack of access to suitable food, water, and shelter are further examples [23,24]. Poor equid welfare can have negative socioeconomic implications for their owners due to the reduced ability to produce income and alleviate household burden, and the social stigma associated with certain diseases [12]. Nor should humans overlook the discomfort, pain, or distress of their equid helpers. This highlights equids’ role in One Health [25] and One Welfare [26]. To overcome these challenges, equine non-governmental organisations and other groups such as research institutions often develop or provide interventions, such as educational and practical training programmes and veterinary services [27]. Establishing the impact of these interventions is important, as this would enable initiatives to be improved or further tailored to the target population and the most effective methods identified. Yet published information focusing on evaluating interventions within the context of working equid welfare is lacking. Upjohn et al., 2014 [27] reviewed and summarised the published literature on the monitoring and evaluation of evidence-based interventions, including educational initiatives, for working equids in LMICs. However, a more recent review has not been conducted, nor one using a systematic search with a structured strategy. Therefore, a scoping review of recent publications assessing the impact of educational interventions in this setting would allow the available evidence to be mapped and could also help inform the design and implementation of future interventions aiming to improve working equid welfare.

## 2. Study Aims and Objectives

This study aimed to use the JBI scoping review methodology to systematically map the literature on two broad topics relating to working equids in low-income, lower-middle-income, and upper-middle-income countries. Throughout this review, these will be grouped and referred to as low- and middle-income countries and abbreviated to LMICs. The first topic was the socioeconomic value of working equids to the livelihoods of their owners and owners’ communities. The second was the impact of educational interventions designed to improve working equid welfare and/or the knowledge, attitudes, and practices of owners and their wider communities relating to working equid care.

### 2.1. Socioeconomic Value Objectives

- This study has created an overview of currently available research on the socioeconomic value of working equids in LMICs, which could be used to inform policy and funding decisions of organisations with an interest in working equids, their owners, and their communities.
- This review has highlighted the socioeconomic value of working equids through their contributions to sustainable development goals.
- Gaps in the literature regarding the socioeconomic impact of working equid ownership have been identified.
- Finally, recommendations have been developed for future studies to aid their discovery and interpretation by researchers, policymakers, and other audiences.

### 2.2. Educational Intervention Objectives

- This study has created an overview of the currently available research on the impact of educational interventions for working equid owners in LMICs on owner knowledge, attitudes, and practices; equid welfare; and any other relevant outcomes.
- This review has highlighted educational intervention strategies that have been successful in achieving their outcomes within this setting.
- Gaps in the literature regarding the evaluation of educational interventions developed for working equid owners have been identified.
- Finally, guidance has been provided for future studies and to inform researchers and other audiences.

## 3. Materials and Methods

### 3.1. Protocol and Registration

The protocols for these two scoping reviews were drafted using the Preferred Reporting Items for Systematic reviews and Meta-Analyses extension for Scoping Reviews (PRISMA-ScR) [28]. This review followed the JBI methodology [29], for which S.L.F had completed the accredited training programme. A.C. had also previously conducted and published a scoping review according to this methodology [30]. The final protocols for each review topic were registered prospectively with Open Science Framework (Socioeconomic Value Registration: https://osf.io/7xb2z/overview?view_only=0db3f8ec115b4a7298b8b52a8b3106c5, accessed on 2 December 2025; Educational Interventions Registration: https://osf.io/mk2xu/overview?view_only=9ceda4cca4ee444cafe247c0dbd47ec8, accessed on 2 December 2025).

### 3.2. Eligibility Criteria

#### 3.2.1. Socioeconomic Value

The eligibility criteria for the socioeconomic value search are described in Table 1. For the purposes of this project, ‘socioeconomic value’ was defined as any impact of working equid ownership on economic factors (including generating and saving money, saving time, and reducing labour) and social factors (including access to education, improving health, building relationships, social status/respect, cultural activities, and leisure activities) and the interactions between these. Publications focusing specifically on the socioeconomic impact of diseases of working equids were excluded, as recent scoping reviews have already been published on this topic [12,17,18].

#### 3.2.2. Educational Interventions

The eligibility criteria for the educational interventions search are described in Table 2. For the purposes of this project, educational interventions included any initiative aiming to improve equid welfare and/or owner/handler safety when working with and caring for equids through the provision of education. To qualify for inclusion, this intervention must be evaluated to assess the impact, if any, on outcomes relating to owner/handler/equine professional/community member knowledge, attitudes, and/or practices relating to equid management, equid welfare, owner injury rates, or socioeconomic factors.

### 3.3. Publication Inclusion

For both topic searches, publications were included if the full text could be obtained from any of the University of Nottingham libraries or e-libraries, as well as from free online Open Access and legal deposit libraries. Grey literature was only included if it reported a relevant study that had not also been published in a peer-reviewed scientific journal and the full text was available. The decision was made to include research published within the last 10 years (from anytime in 2014 onwards) to build on the educational interventions review conducted by Upjohn et al., 2014 [27] and increase the likelihood that socioeconomic information was relevant and up to date.

### 3.4. Information Sources

To identify potentially relevant publications, the following electronic databases were searched on 24 April 2024:

- CAB Abstracts (Ovid): 1973–present;
- Ovid MEDLINE: 1946–present;
- Embase (Ovid): 1974–present;
- Web of Science (Core Collection): 1900–present;
- International Bibliography of the Social Sciences (IBSS): 1951–present.

### 3.5. Search Strategy

The search strategy was drafted by the research team, and feedback was provided by an experienced University of Nottingham librarian. The same search strategy was used for both review topics. A summary of the search strategy for CAB Abstracts is shown below. The full search strategy used for each database can be found on Open Science Framework (https://osf.io/7xb2z/files/tdnpe?view_only=0db3f8ec115b4a7298b8b52a8b3106c5, accessed on 2 December 2025). A filter was applied to limit the search to publications from or about LMICs. This filter was based on the Cochrane Effective Practice and Organisation of Care (EPOC) LMIC filter from February 2023 [32] and was adapted slightly based on some of the suggestions from the School of Health and Related Research (ScHARR) LMIC filter [33]. These adaptations included expanding the database fields that were searched. Additionally, as the focus of this review was only on studies published from the last 10 years, previous LMICs that became categorised as high income before 2014 and have since stayed in this category, according to data from The World Bank 2024 [34], were removed from the filter. Data published by The World Bank 2024 [34] were also assessed to identify any changes in country classifications since the publication of the filter that would require additional countries to be added; no additions were needed.

Forwards and backwards citation searching were undertaken for all publications identified and included through the database searches. This was carried out on 22 August 2025 using citationchaser [35]. Any publications not found on citationchaser had their reference lists manually screened during backwards citation searching. For forwards citation searching, Google Scholar and Web of Science were searched to identify publications that had cited them. This process of citation searching was repeated with new publications which met eligibility criteria until no further new publications were identified.

### 3.6. CAB Abstracts Search Strategy Summary

(((exp horse/or exp donkey/or exp mule/or (equus or equid*2 or horse*2 or equine* or donkey* or pony or ponies or mule or mules or hinny or hinnies or ass or asses).mp.) and (cart* or draught* or draft* or pack*3 or plough* or plow* or transport or traction* or carriage*1 or “horse drawn” or working or labour*).mp.)

OR

(“work* equid*” or “work* horse*” or “work* mule*” or “work* donkey*” or “work* hinn*” or “work* ass” or “work* asses” or “work* pony” or “work* ponies” or “work* equine*” or carthorse* or cart-horse*).mp.

OR

((exp working animals/or exp draught animals/or LL060.cc.) and (equus or equid*2 or horse*2 or equine* or donkey* or pony or ponies or mule or mules or hinny or hinnies or ass or asses).mp.))

AND

[LMIC Filter]

AND

[Publication date 2014–2024]

### 3.7. Study Selection

To validate the screening approach, the same 50 titles and abstracts from a pilot search were reviewed by two researchers. The results were discussed to determine whether it was necessary to amend the inclusion and exclusion criteria before the full screening for this review. As a result, the following specifications were added to the criteria to increase clarity: socioeconomic value publications must report a measure of socioeconomic impact in the methods and results; for educational interventions, publications must sufficiently describe the intervention and methods of evaluation; for both topics, conference contributions must be >300 words. References were downloaded into EndNote X9 (Thomson Reuters), and duplicates were removed before being imported into Covidence (an online collaborative systematic review tool) [36]. Two researchers independently reviewed titles and abstracts for agreement with the eligibility criteria, with any ambiguous publications being retained for the next stage where the full text was reviewed. Any disagreements were discussed until a consensus was reached. If a consensus was not reached, then the opinion of a third reviewer was enlisted. This process was repeated during the review of full texts. Each publication was screened to assess eligibility to be included in the socioeconomic value review, educational interventions review, or both. Where a publication could be excluded for multiple reasons, it was listed as excluded by the first reason that became apparent.

### 3.8. Charting Process and Synthesis of Results

Publications which met the inclusion criteria for each search were read in full and assessed so relevant information could be extracted and charted. This task was split between the authors, who each independently charted their allocated studies into pre-agreed forms. Socioeconomic value studies and educational intervention studies were charted separately. The study characteristics charted were authors, year published, study methods, main study aims, country of focus, income classification of country (according to their status for the 2024 fiscal year [34]), human population, equid species, equid use, and number of equids. The key findings were charted in separate tables for both journal articles and reports; conference papers were not charted due to providing limited detail. For socioeconomic studies, the socioeconomic measures used were also charted. For educational intervention studies, the intervention strategy and evaluation measures used were also charted.

## 4. Results

### 4.1. Selection of Evidence Sources

**Figure 1:**
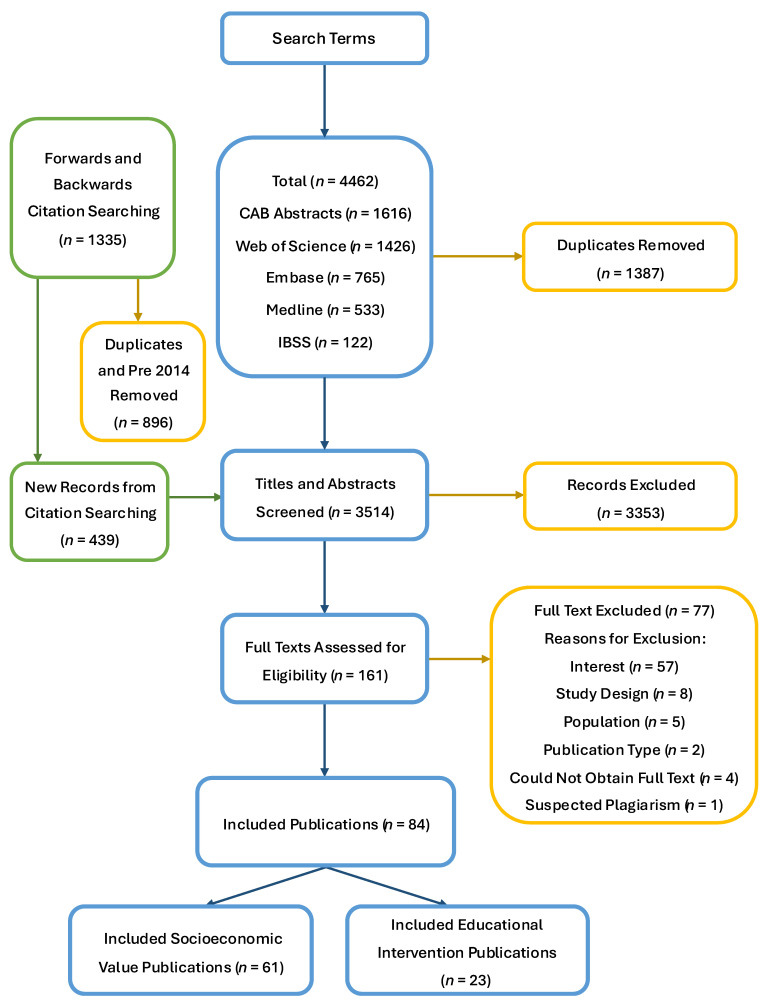
Study selection process for both the socioeconomic value and the educational interventions scoping reviews.

A total of 4462 publications were identified through the five database searches. After the removal of duplicates, 3075 remained and were screened by title and abstract, after which 139 studies were retained for full-text screening, and 67 met the inclusion criteria. Through forwards and backwards citation searching, after the removal of duplicates and sources published before 2014, 439 additional publications were identified. After title and abstract screening, 22 underwent full-text review, and 17 met the inclusion criteria. Therefore, a total of 84 studies met the eligibility criteria and were included in either of the review topics. This was 61 for the socioeconomic value scoping review and 23 for the educational interventions scoping review. Of the full texts screened, 12 were written in a language other than English and were translated to English using DeepL before screening [31]. Most studies excluded during full-text screening were due to them not focusing on the topic of interest (*n* = 57). Four publications were excluded due to inaccessible full texts. One publication was excluded as it was assessed by reviewers to contain data and text plagiarised from an earlier published study, which was included in the review. The full list of exclusion reasons can be found in Figure 1, along with the screening process for both the socioeconomic value and educational interventions reviews.

Broad search terms were applied due to the inconsistent terminology used across publications. Several publications identified through citation searching did not contain work-related terms in the title, abstract, keywords, or other database fields searched, and one did not include equid-related terms. A range of terms were also used to describe educational interventions and the socioeconomic contributions of working equids. The term “livelihood(s)” was most commonly used when describing the socioeconomic value of working equids, appearing in the titles and/or abstracts of 39/61 publications. Other terms used included “socioeconomic”, “economic”, “income”, and “social” (or associated variations). Five studies did not include any of these terms. Of the included educational intervention studies, the initiatives introduced were often described using terms other than “intervention”. However, all but one of the included publications contained at least one of the following terms (or associated variations) within the title or abstract: “intervention”, “initiative”, “education”, or “training”. In addition, five studies did not include the country of focus within these commonly searched database fields.

### 4.2. Synthesis of Results

#### 4.2.1. Socioeconomic Value

The key study characteristics of the 61 eligible publications are presented in Table 3 and split by publication type. Forty-seven publications were journal articles, twelve were conference contributions, and two were reports from non-governmental organisations (NGOs). All but two of the conference contributions were from a single conference, the 7th International Colloquium on Working Equids, held by World Horse Welfare in 2014 [37]. Most studies were conducted in one country only, while three were conducted in multiple countries. Studies were most commonly conducted in Ethiopia (15, including 1 multi-country study), followed by India (11, including 2 multi-country studies), and Kenya (10, including 2 multi-country studies). Other countries were represented in a maximum of four studies. Lower-middle-income countries were most represented (34, including multi-country studies). All but three included studies were published in English. These were conducted in Brazil and published in Portuguese [38,39,40]. Forty-two studies focused on only one species of equid, while thirteen covered horses, donkeys, and mules. Donkeys were the most represented species across all studies (45/61). Four of the conference contributions did not specify the species of working equid studied. Working equids were used for income generation, saving time/labour/money, and cultural purposes. In the majority of studies, working equids were used for multiple purposes and were most commonly described as being used for transport of people, goods, or other items (40/61); cart pulling (24/61); agriculture (24/61); pack carrying (21/61); or domestic tasks (17/61). The human population studied was typically working equid owners or users, and some studies also included other stakeholders, such as veterinary professionals, animal health care providers, local community members, tourists, and children. Studies employed a range of methods, but social science methods such as surveys, interviews, and focus groups were common. Of the 47 peer-reviewed journal articles, 35 were published from 2020 onwards. The quality and clarity of the included studies were variable.

The key results from the 47 journal articles and two reports relating to the socioeconomic value of working equids are presented in Table 4. A wide range of measures were used to capture the socioeconomic impact working equids had on their owners and owners’ wider communities, with some being measured quantitatively and others qualitatively. The most commonly used socioeconomic measure was impact on income generation, with 46/47 publications reporting that income generation was an important contribution of working equids. Sixteen publications reported that for the majority of their participants, their sole or primary income was generated through their working equid. Not all studies collected data on the primary source of income, so this cannot be directly compared between all sources. However, a further 30 studies emphasised the importance of the income generated both directly and indirectly through working equids, which was often essential in supporting participants’ livelihoods, even if they were not stated as the primary income source. Fourteen publications reported the mean, median, or range of actual incomes equid owners generated from their equid(s), with the range of average monthly incomes across all studies being USD 18.15 [41]–664.70 [42] (daily incomes were multiplied by 30 and annual incomes were divided by 12 to approximate monthly income). Ten publications reported the mean or median income (as opposed to just the range), and the mean average monthly income generated from equid use across these studies was USD 217.26. However, income figures are not directly comparable across studies due to differences in how income was measured and reported, inflation over time, and varying purchasing power across countries. Additionally, 14 of the 47 publications discussed the crucial role working equids had in reducing physical labour. This was described as leading to improved health for their owners and saving time which could be spent on other activities, such as income generation or social interests. Twelve publications highlighted that working equids were especially valuable to women. Equids were often described as playing essential roles in women’s lives and contributing to female empowerment. Thirteen publications reported that owning a working equid could increase an owner’s social status within their community.

**Table 3:** Characteristics of studies identified in a scoping review investigating the socioeconomic value of working equids to their owners and communities. Country income status according to The World Bank 2024 [34].

Author and Year	Main Study Aims	Human Population	Equid Use	Equid Species and No.	Study Methods	Country and Income Status
**Journal Articles**						
Abdifatah Ahmed et al., 2023 [43]	To (1) establish the influence of donkey owners’ perceptions on donkey welfare, (2) determine the influence of donkeys’ contributions to owners’/user’s livelihood on donkey welfare, and (3) asses the influence of owner/user training on donkey welfare	156 donkey owners/users	Cart pulling	>288 donkeys (exact no. not specified)	Cross-sectional survey	Somalia (Low)
Alam et al., 2015 [44]	To study the socioeconomic status of horse keepers and income from horse rearing	200 horse-owning households	Cart pulling	Horses No. not specified	Structured interviews	Bangladesh (Lower-Middle)
Asfaw and Tadesse 2020 [45]	To study the economic contribution of carthorses to livelihood of their owners	200 cart horse owners	Cart pulling; pack carrying	135 horses	Semi-structured questionnaires; physical exam of horses	Ethiopia (Low)
Asrat et al., 2019 [46]	To investigate cart pulling donkeys’ contribution to their owners’ livelihoods, and the impact of donkey foot related problems	369 donkey owners	Cart pulling	369 donkeys	Structured Interviews; clinical exams	Ethiopia (Low)
Asteraye et al., 2024 [47]	To assess population dynamics, distribution, biomass, and economic value of equids in Ethiopia	7 knowledgeable elders, 10 key informants	Cart pulling; transportation	Horses, donkeys, mules No. not specified (census data)	Analysis of census data; structured Interviews	Ethiopia (Low)
Avornyo et al., 2015 [41]	To assess the contribution of donkeys to food security for their owners (physical and economic access to sufficient nutritious food)	100 donkey owners	Cart pulling (for transportation of water, building materials, agricultural material, produce); agriculture (ploughing); manure production	119 donkeys	Questionnaires; interviews	Ghana (Lower-Middle)
Badmos et al., 2019 [48]	To consider donkey keeper and draught operators’ perceptions of donkey welfare and management issues in relation to economic uses	200 donkey farmers/owners	Pack carrying; domestic tasks; agriculture	Donkeys No. not specified	Semi-structured questionnaires	The Gambia (Low)
Barbosa et al., 2020 [38]	To assess the nutritional, health and reproductive management of draft horses. To assess the socioeconomic conditions of the region’s cart drivers and provide guidance on animal management	23 cart drivers	Cart Pulling	11 donkeys; 9 horses; 8 mules	Structured interviews	Brazil (Upper-Middle)
Carder et al., 2019 [49]	To explore the potential impact of the donkey hide trade on small holder farmer’s livelihoods	421 current and previous donkey owners surveyed; 33 focus groups (5–7 per group) with farmers, transporters, business owners; 48 key informants (government representatives) interviewed	Transport of water; domestic tasks; agriculture; renting out to others; transport of passengers; dowry asset	Donkeys No. not specified	Surveys; focus groups; interviews	Kenya (Lower-Middle)
Cousquer et al., 2023 [50]	To understand how muleteering has emerged in the region, to document working life, husbandry, and health and welfare concerns for mules	90 owners surveyed, ethnographic study numbers unclear	Agriculture; construction; mountain tourism	88 mules; 2 donkeys	Mixed methods: Ethnographic walking and reading, survey/structured interview, home visit and clinical examination	Morocco (Lower-Middle)
de Klerk et al., 2020 [51]	To understand the social and economic impact the use of a horse and cart on an individual, their household, the surrounding community and the horse itself, and understand the spatial extent to which the cart horses work	100 cart horse drivers	Cart pulling	163 horses	Questionnaire	South Africa (Upper-Middle)
Desta 2023 [52]	To report the diverse use values of equines and their current population status	10 knowledgeable farmers	Agriculture; transport; pack carrying; cultural events	Horse; donkey; mule No. not specified (census data)	In-depth interviews; analysis of livestock census data	Ethiopia (Low)
Geiger and Hovorka 2015 [53]	To explore the lives of donkeys and donkey-human relations	100 donkey owners	Cart pulling; domestic tasks; agriculture	100 donkeys	Semi-structured interviews; welfare assessments	Botswana (Upper-Middle)
Geiger et al., 2020 [54]	To identify the personal, social, and broader economic value of donkeys to rural, peri-urban, and urban households	20 donkey owners/users, 10 key informants	Cart pulling; pack carrying	Donkeys No. not specified	In-depth interviews	Ethiopia (Low)
Geiger et al., 2021 [55]	To investigate the differences in donkey owners’ uses and beliefs of donkeys and donkey welfare between rural and urban locations	28 donkey owners—15 rural, 13 urban	Pack carrying; domestic tasks; agriculture; transport; rubbish collection; cart pulling	161 donkeys	Questionnaires; welfare assessments	Ethiopia (Low)
Geiger et al., 2023 [56]	To investigate donkeys’ multidimensional contributions to their human co-workers’ lives	137 human donkey co-workers	Domestic tasks; agriculture; transport; pack carrying; construction; rubbish collection; cart pulling	Donkeys No. not specified	Workshops using participatory rural appraisal and appreciative inquiry	Ethiopia (Low)
Geiger 2023 [57]	To explore human-donkey relationships and how gendered divisions of labour manifest across species lines	20 donkey owners; 10 key informants	Pack carrying; domestic tasks; agriculture; construction; rubbish collection	Donkeys No. not specified	Semi-structured interviews; participatory workshops	Ethiopia (Low)
Gelaye and Fesseha 2020 [23]	To assess the socioeconomic importance and constraints of equids in Central Ethiopia	150 equid owners	Pack carrying; cart pulling; cultural and religious events; agriculture; renting out; breeding and selling	205 horses; 232 donkeys; 2 mules	Cross-sectional survey (structured interview questions) and an observational study	Ethiopia (Low)
Gichure et al., 2020 [8]	To determine the benefits of keeping donkeys and associated production challenges under a smallholder farming system	13 focus groups of 8–12 donkey owners	Transport; Manure production; breeding; agriculture	Donkeys No. not specified	Focus groups	Kenya (Lower-Middle)
Gichure et al., 2020 [58]	To determine farm level factors associated with household incomes for farms that keep donkeys in a smallholder farming system	354 smallholder farming households keeping donkeys	Cart pulling; transport; domestic tasks; agriculture	1040 donkeys	Semi-structured interviews	Kenya (Lower-Middle)
Gina and Tadesse 2015 [59]	To examine the role of working animals in livelihoods and food security	120 working animal owners (51 donkey owners)	Cart pulling; transport; pack carrying; renting out	Donkeys No. no specified	Semi-structured interviews; focus groups	Ethiopia (Low)
Gupta et al., 2017 [60]	To document the physical, biometric indices, health and managemental issues of working donkeys for future improvement and proper management of working equids	Owners/handlers of brick kiln donkeys No. not specified	Brick Kiln	98 donkeys	Physical examination and presumably discussions with owners/handlers but not described in methods	India (Lower-Middle)
Gursoy 2020 [61]	To explore sustainable tourism and transport, specifically the human-animal relationship, by taking horse-drawn carriages as objects of inquiry	37 stakeholders (including carriage drivers, vets, tourists, local inhabitants)	Tourist carriage rides	Horses No. not specified	Semi-structured interviews	Türkiye (Upper-Middle)
Kithuka et al., 2025 [62]	To assess the role of environmental and human factors on the welfare of working donkeys	1059 donkey owners	Pack carrying; cart pulling; agriculture; water transport; renting out	1059 donkeys	Semi-structured interviews	Kenya (Lower-Middle)
Koko and Shuiep 2016 [42]	To assess the socioeconomic value of rearing donkeys, and to investigate the frequency of different ecotypes of donkey based on phenotypic characteristics	105 donkey owners	Cart pulling; transport of goods and people; agriculture	105 donkeys	Structured interviews	Sudan (Low)
Kubasiewicz et al., 2022 [5]	To investigate the links between poverty, equid ownership and equid welfare in the brick kilns of Ahmedabad, India	32 donkey owners; 5 thekedars (supervisors); 6 non-owner workers	Brick Kiln	220 donkeys	Semi-structured interviews; livelihood questionnaires; welfare assessments (EARS)	India (Lower-Middle)
Kubasiewicz et al., 2023 [63]	To (1) describe the welfare of donkeys owned under conditions of debt-bondage, examine the links between owner and donkey behaviour, and outline the living conditions of both donkeys and humans working in brick kilns; (2) explore the experience of debt-bondage, compare migration trends to those of non-donkey-owning workers, and assess impacts on their children’s education	32 donkey owners; 5 thekedars (supervisors); 6 non-owner workers; 3 kiln owners	Brick Kiln	220 donkeys	Semi-structured interviews; livelihood questionnaires; welfare assessments (EARS); observational assessments	India (Lower-Middle)
Kubasiewicz et al., 2024 [64]	To (1) outline the role of mules in supporting resilient communities in the remote mountains and identify the role of mules in meeting the Sustainable Development Goals; (2) explore the relationships between equid handling experience and equid welfare; (3) provide insight into the mindset of key informants in the face of both current risk exposure, and long-term systemic change from development	Livelihood surveys: 23 mule owners and drivers, 26 non-mule owning community members Interviews: 27 mule owners and drivers, 28 non-mule owning community members	Pack carrying	127 mules	Livelihood surveys; semi-structured interviews; welfare assessments (EARS)	Nepal (Lower-Middle)
Maggs et al., 2021 [6]	To examine the role of donkeys in northern Ghana and how donkeys contribute to livelihood outcomes, especially for women and children	262—combination of adult and child donkey owners and non-donkey owners	Agriculture; transport; cart pulling; domestic tasks; construction; community events	Donkeys No. not specified	In-depth interviews; focus groups; surveys; time budgets	Ghana (Lower-Middle)
Maggs et al., 2023 [65]	To understand the utilitarian value donkeys provide to poor small holder farmers, especially women, in their efforts to make a living in rural northern Ghana	Questionnaire—28 donkey owners and 10 children, 8 non-donkey owners and 10 children; interviews—6 donkey owners, 4 non-donkey owners; focus groups—54 donkey owner children	Agriculture; transporting goods; domestic tasks	Donkeys No. not specified	In-depth semi-structured interviews; questionnaires; focus groups	Ghana (Lower-Middle)
Merridale-Punter et al., 2024 [66]	To (1) describe the work equipment used by working equids; (2) understand the implications of harnessing practices to both animals and the community; (3) describe the knowledge, attitudes and practices of working equid users in regard to work equipment	368 cart drivers surveyed; 87 participated in focus groups—77 working equid owners and drivers, 9 harness/cart makers, 1 vet	Cart Pulling (for taxi transport, goods, or water)	243 horses; 122 donkeys; 3 mules	Mixed methods: survey, questionnaire and focus groups	Ethiopia (Low)
Merridale-Punter et al., 2024 [67]	To (1) explore the specific One Health links between working equids and their female users through a collection of personal stories; (2) explore the interconnectedness of those links using a systems thinking approach	10 female working equid users	Domestic tasks; agriculture; transport (of people, produce, food, water); other income generation	Horses; donkeys No. not specified	Semi-structured interviews	Ethiopia (Low)
Narayanan 2024 [68]	To politicise the emotional, physical, and psychological suffering of animals in coercive labour, and challenge the anthropocentric focus of the development and antipoverty praxis	Ethnographic visits to ~100 kilns Interviews: six vets, 7–10 animal owners, manager from each kiln	Brick kiln	Horses; donkeys; mules No. not specified	Ethnography; semi-structured interviews; direct observations	India (Lower-Middle)
Nguekeng et al., 2022 [69]	To set baseline information on the characteristics of donkey husbandry, including the socioeconomic and technical characteristics of donkey farming	149 donkey owning farmers	Breeding (to be used for transport and agriculture)	Donkeys No. not specified	Mixed methods: interviews, observations and questionnaire	Cameroon (Lower-Middle)
Oduori et al., 2025 [70]	To assess the economic impact of the donkey skin trade on donkey-dependent women, their families, and communities	Questionnaires: 171 women Interviews: 17 women	Transport (of water, produce, firewood, other items for commercial purposes); domestic tasks; manure production; milk production	Donkeys No. not specified	Cross-sectional questionnaires; key informant interviews	Kenya (Lower-Middle)
Onono and Kithuka 2020 [71]	To determine benefits of keeping donkeys, challenges facing donkey farmers, and how to streamline the supply of medicines for treatment of donkeys	156 donkey owners and users; 87 animal health service providers and agro-vets	Transportation (of water, produce, animal feed, firewood, construction materials, manure); renting out; agriculture	Donkeys No. not specified	Semi-structured questionnaires	Kenya (Lower-Middle)
Rink and Crow 2021 [72]	To explore experiences of working horses and cart drivers, including mobility and livelihoods	1 cart driver	Cart pulling (rubbish collection)	1 horse	Ethnographic study with interviews and observations	South Africa (Upper-Middle)
Sangioni et al., 2016 [39]	To assess the welfare conditions of draught horses and to verify the socioeconomic profile of their respective owners	123 owners	Cart pulling	191 horses	Cross-sectional study: animal clinical assessments and owner questionnaire	Brazil (Upper-Middle)
Shah et al., 2019 [73]	To assess the role and welfare of cart donkeys used in waste management, and understand the challenges faced; to aid the development of interventions	200 owners, 50 households, 14 key informants	Cart pulling: waste removal	204 donkeys	Mixed methods: owner interviews and SEDWAT animal welfare assessments	Pakistan (Lower-Middle)
Tuaruka and Agbolosu 2019 [74]	To determine whether there were differences in the production and management of donkeys in communities in Bukpurugu/Yunyoo district	100 donkey owners	Cart-pulling; transport (of water, produce, farm implements); agriculture	144 donkeys	Semi-structured interviews; physical measurements and observations of donkeys	Ghana (Lower-Middle)
Teixeira et al., 2022 [40]	To describe cart drivers’ general, socioeconomic, and occupational characteristics, and the way they manage their horses	38 owners	Cart pulling	38 horses	Structured interviews	Brazil (Upper-Middle)
Vasanthakumar et al., 2021 [11]	To investigate (1) how working equids contribute to women’s livelihoods, (2) the roles women have in caring for their equids, (3) the opportunities women have to acquire new knowledge about their equids, (4) whether women find existing training programmes helpful and accessible, and (5) the areas of equid care on which they would like more information	34 female equid owners	Pack carrying; domestic tasks; agriculture; transport; tourism; breeding	Horses, donkeys, mules No. not specified	Structured interviews	Guatemala (Upper-Middle)
Wani et al., 2021 [75]	To investigate the socioeconomic status of ponywallas associated with ecotourism in the Kashmir valley	200 ‘Ponywallas’ (horse owners)	Transport; tourism	Horses (ponies) No. not specified	Semi-structured interviews	India (Lower-Middle)
Watson et al., 2020 [76]	To (1) outline the complexities of the lives of the poorest in India, (2) explore the Hindu caste and Scheduled Tribe systems, and (3) examine how cultural “blind spots” create challenges for NGOs attempting to target donkey welfare	37 donkey owners	Brick kiln	219 donkeys	Mixed methods: livelihood survey, welfare assessment using EARS tool, semi-structured interviews	India (Lower-Middle)
Watson et al., 2022 [77]	To (1) investigate the lives of equids walking mountain trails in Nepal; (2) document and discuss their transient existence and the challenges they face	24 mule owners/drivers, 1 mule trader, 2 veterinary technicians	Pack carrying	166 mules	Mixed methods: semi-structured interviews, livelihood surveys, welfare assessments using EARS tool	Nepal (Lower-Middle)
Watson et al., 2023 [78]	To understand the scale of the challenges facing working equids operating on mountain trails in Nepal	24 mule owners	Pack carrying	166 mules	Mixed methods: semi-structured interviews, livelihood surveys, welfare assessments using EARS tool	Nepal (Lower-Middle)
Wild et al., 2021 [21]	To assess the impact of the COVID-19 pandemic on the working equid community	1530 working equid owners/users	Pack carrying; domestic tasks; agriculture; transport; rubbish collection; cart pulling	Horses, donkeys, mules No. not specified	Cross-sectional survey	Cambodia, Haiti, Honduras, Lesotho, Nepal, Nicaragua, Senegal, Zimbabwe (Lower-Middle); Colombia, Costa Rica, Guatemala, Mexico, South Africa (Upper-Middle); Panama (High—last Upper-Middle in 2022 fiscal year)
**Reports**						
Valette 2014 [13]	To explore the contributions of working horses, mules, and donkeys to the lives of women	Women who work with equids—Ethiopia: 58, Kenya: 53; India: 88, Pakistan: 60	Transport; domestic chores; manure; agriculture; cart pulling; pack carrying, brick kilns, rubbish collection	Horses; donkeys; mules No. not specified	Focus groups and interviews	Ethiopia (Low); Kenya, India, Pakistan (Lower-Middle)
Valette 2015 [79] *	To understand the economic contributions of working donkeys, horses, and mules to household incomes	Kenya: 254 participants—donkey owners and users, and control group of taxi operators India and Pakistan: no. not specified	Cart pulling; transport; pack carrying; renting out; milk production; agriculture	Horses; donkeys; mules No. not specified	Household economy approach—including interviews/questionnaires	Kenya, India, Pakistan (Lower-Middle)
**Conference Papers**						
Abbas 2014 [80]	To gather economically marginalised women’s views and experiences of the role of working donkeys in their lives and document how women manage their donkeys	85 women	Domestic Tasks; pack carrying, cart pulling; transport; other income generation	Donkeys No. not specified	Interviews and focus groups	Pakistan (Lower-Middle)
Asmamaw et al., 2014 [81]	To identify the major uses of equids and their contribution to household livelihoods	50 households interviewed, 10 focus groups with key informants	Pack carrying; cart pulling; domestic tasks; transport; renting out; selling; social events	Horses; donkeys; mules No. not specified	Mixed methods cross-sectional study: semi-structured interviews and focus groups	Ethiopia (Low)
Bekele et al., 2014 [82]	To assess the socioeconomic impact of epizootic lymphangitis on the livelihood of cart mule owners	109 mule owners	Cart pulling	Mules No. not specified	Questionnaires, interviews, focus groups	Ethiopia (Low)
Doumbia 2014 [83]	To show the role of working donkeys in the livelihoods of the population in the villages in Segou, Mali	1044 families; financial data from 350 owners	Cart pulling; other unspecified roles	1754 donkeys	Questionnaires	Mali (Low)
Kandpal et al., 2014 [84]	To investigate the contribution of equids in the livelihoods of the poor, marginalised communities engaging in brick transport	200 owners	Brick kiln (cart pulling and pack carrying)	Species and no. not specified	Mixed methods: structured interviews, focus groups, activity schedules, resource and mobility mapping, credit analysis	India (Lower-Middle)
Kendagor and Njoroge 2014 [85]	To determine the contribution of donkeys to the livelihoods of a marginalised group of women	15 members of a women’s group	Transport of water, food, and firewood	Donkeys No. not specified	Focus groups	Kenya (Lower-Middle)
Lane 2015 [86] ^†^	To evaluate household demographics and wealth, respondent health, child anthropometry, and working equine health	70 owners; 20 children of owners	Pack carrying for agricultural goods	107 horses; 18 mules; 7 donkeys	Cross-sectional verbal survey; welfare assessments; analysis of growth data of children	Nicaragua (Lower-Middle)
Mwasame et al., 2019 [87]	To provide empirical evidence on the economic and non-economic value of donkeys to human livelihood	134 donkey owners; 121 non-owners	Transport (including water, building materials, produce, fertiliser); domestic tasks; renting out	Donkeys	Cross-sectional survey	Kenya (Lower-Middle)
Rodriguez Rodas and Perez 2014 [88]	To understand the status and relationship between equids, owners, and communities	325 owners	Transport of goods	Species and no. not specified	Questionnaire and correlation analysis to generate community profiles	Guatemala (Upper-Middle)
Warboys et al., 2014 [89]	To gather information and provide an insight into the general public’s knowledge and perception of the role of working equids in and around the Choluteca area; and the importance of this role to the local economy	106 owners	Firewood collection; other	Species and no. not specified	Cross-sectional study: structured interview and questionnaire	Honduras (Lower-Middle)
Zaman et al., 2014 [90]	To investigate and quantify the financial contribution of equids to livelihoods of households using the Household Economy Approach analytical tool	Community leaders and participants from three wealth groups from four villages, total no. participants not specified	Brick kilns; transporting goods and people; agriculture	Species and no. not specified	Interviews; household economy approach analysis	India (Lower-Middle)
Zaman et al., 2014 [91]	To explore the role of working equids in the lives of women in India, facilitate discussion on socioeconomic issues relating to women’s use of equids and document their perspectives on equid use and care	78 women	Brick kiln; other goods transport; domestic tasks	126 horses, donkeys, and mules	Interviews and focus groups	India (Lower-Middle)

* Data taken from Household Economy Approach case study boxes. ^†^ Relevant section: ‘Understanding the Association Between Working Equid Health and Human Health in Rural Nicaragua’.

**Table 4:** Key findings from studies identified in a scoping review investigating the socioeconomic value of working equids to their owners and communities.

Author and Year	Socioeconomic Measures	Key Findings
**Journal Articles**		
Abdifatah Ahmed et al., 2023 [43]	Participant demographics Income generation from equids; impact of equids on financial and food security	Participants were from Somalia and were all male, with three quarters educated to primary level and the rest having no formal education. When asked how donkeys contributed to their livelihoods, 24% respondents said that donkeys contributed to an improved income, 22% said that donkeys contributed to food security, 21% said that donkeys were a source of money, 17% said that donkeys are a source of financial capital, and 16% said that donkeys contributed to increased savings.
Alam et al., 2015 [44]	Participant demographics Primary occupation; secondary occupation; monthly income; source of horse and income from horse (through cart-pulling)	The only income for many horse owning households in Bangladesh was from their horse cart-pulling; for 88%, it was their primary occupation. Moreover, 54% were landless and 85% illiterate. They worked an average of 20 days/month, 7–8 h/day. Monthly income from horse use ranged from 3000 to 20,000 BDT/month (USD 39.14–260.96 *), with 66% earning 5000–8000/month (USD 65.24–104.38 *). Horse keepers earnt more during the rice and fruit seasons, and younger participants earnt more.
Asfaw and Tadesse 2020 [45]	Participant demographics Daily income	Income from horse work supported cart horse owners’ family livelihoods in Ethiopia. In the studied population, 67% had at least an elementary school level of education; 81% had 1–6 other family members, and 60.5% had 1–2 children at school; 44.5% had 2 horses; 27.5% had 1. Horses were commonly used >8 h/day (42%) for 5–7 days/week (99%) to transport loads of 300–700 kg (66%). Daily income ranged from 50–20 ETB/day (USD 2.47–5.94 *); 59% earnt 50–100 ETB/day (USD 2.47–4.95 *).
Asrat et al., 2019 [46]	Participant demographics Annual income from donkey use; annual monetary loss from donkeys suffering foot problems	In this study, 94% of donkey owners in Ethiopia were fully dependent on their cart donkey for their household’s livelihood; 89% owned 1 or 2 donkeys; 95% had completed at least elementary school. Participants worked with their donkeys 5–7 days/week for an average of 8 h/day. Average daily income was ETB 124 (range of ETB 32–360). Average annual income was ETB 29,760 (approx. USD 1488), average annual net contribution from the donkey was ETB 12,563 (USD 626.80). Moreover, 38% donkeys had a foot problem. Owners were estimated to lose an average of ETB 2232/year (range ETB 1240–7740) due to donkeys not being able to work because of foot problems. Culling due to foot problems caused significant financial loss; the cull rate in the study was 1.4%.
Asteraye et al., 2024 [47]	Number of horses, donkeys and mules; purpose, price and rental value of animals Biomass of equid population; stock monetary value; equid service value	Equids are important in transport and agriculture in Ethiopia and contribute significantly to the national economy. The per capita number humans to equids was 0–0.52 for donkeys, 0–0.13 for horses, and 0.02 for mules. Equids represented 10% of the total livestock biomass, 3.1% of the total livestock monetary value (USD 1229 million). The service value of transportation and draft was estimated as USD 1198 million, which was up to 1.2% of the country’s national GDP.
Avornyo et al., 2015 [41]	Income generation Food security	Donkeys in Ghana earnt owners a mean annual income of USD 217.78, which contributed 19% of their total income (the second largest contributor after crops). This was broken down into a mean of USD 110.06 through transporting loads, USD 70.56 from being hired out, USD 28.27 from manure sale, and USD 8.89 from ploughing (due to low numbers that used donkeys for this purpose). Donkey use could provide an estimated average profit of USD 707.50 over five years, after accounting for the cost of maintaining the donkey and cart. Female-headed households had lower levels of food security then male-headed households and were more likely to use donkeys to increase food security.
Badmos et al., 2019 [48]	Participant demographics Use of donkeys	Owners in Gambia used their donkeys for commercial activities (59%), carrying water or other domestic purposes (34%) and farming activities (7%).
Barbosa et al., 2020 [38]	Participant demographics Education level Working hours and load Income generation	Cart drivers’ main or sole source of income was derived from horse traction, carrying mainly construction materials and rubble. In the studied population, 57% of participants were over 41 years old, with 57% having incomplete primary education with 91.3% starting work before legal adulthood; 52% learned their profession from their own experience; 52% were working 9 h or more with 61% for 5 or more days a week; 69% reported they loaded their equids 101–450 kg.
Carder et al., 2019 [49]	Participant demographics Labour reduction; income generation; food security; independence; impact of loss of donkeys due to skin trade	Household survey respondents in Kenya: 82% smallholder farmers, of these 93% reared donkeys. The donkey hide trade had led to an increase in the sale and theft of donkeys in the area, decreasing the local donkey population. This reportedly affected community members with disabilities (who rely on donkeys for daily chores), with 63% now relying on voluntary assistance from others to complete chores. Additionally, 53% respondents reported increased spending on transport of farm produce, water, and firewood, and 43% reported an increase in time spent carrying water and firewood. Focus groups in Kenya: 66% farmers; 22% reported not wanting to sell their donkeys but had to due to a need for instant cash for their families. Most focus group and interview participants reported an increase in donkey, theft and decrease in food and financial security of donkey owners associated with the skin trade. Some felt the donkey skin trade had a positive impact, meaning they could easily sell their donkey to raise instant cash if needed, e.g., to pay children’s school fees. However, 63% reported they previously earnt money from donkeys to raise money for school fees, but now were unable to pay these fees due to a reduction in donkeys. Moreover, 56% focus group participants felt that loss of donkeys pushed back progress for women who rely heavily on them for their livelihoods.
Cousquer et al., 2023 [50]	Use of mules	Mules were often used in tourism, providing revenue for the families that owned them in the High Atlas Mountains of Morocco. The main income was from summer tourism, and money saved from this was used for subsistence during the winter. Additional uses were in transportation of building material, manure, and agricultural produce.
de Klerk et al., 2020 [51]	Use of horses; contribution to income; contribution to family and surrounding residents	Working cart horses in Cape Town, South Africa was the primary source of income for 89% of owners, and also helped support their families and surrounding community. The main use was transporting scrap metal (46%) and garden refuse (32.3%). Daily earning from horse-related income varied between ZAR 0–900 (USD 0.00–65.65 *), with 60% earning between ZAR 0–300 (USD 0.00–21.88 *). Participants’ mean daily income was ZAR 287.07 (USD 20.94). Participants supported a mean of 2.9 children, 2.2 family adults, 1.0 employees, and 1.1 non-related person.
Desta 2023 [52]	Use of equids	Famers in Ethiopia described their use of equids in agriculture, community events such as festivals, weddings and funerals, transportation, manure production, and use of equid-derived products.
Geiger and Hovorka 2015 [53]	Participant demographics Use of donkeys	Donkey owners in Botswana described the economic and social contributions of their donkeys, particularly around assistance with household tasks. 100% of participants used their donkeys for transporting fuel or water, 97% used them for ploughing, and 87% used them for riding to move cattle. Moreover, 24% of people described their donkeys as family members, contributing within the household and homestead; 27% described their utilitarian value (foodstuff, plough, vehicle); 32% described assistance and support (income provision, food provision, rest and spiritual guidance). The labelling of donkeys as ‘companion animals’ rather than ‘food animals’ was described as making them subordinate to cattle and resulting in marginal positioning. Impacts described were a lack of concern from government and policies, reduced access to veterinary resources and treatments, and a low market value for donkeys.
Geiger et al., 2020 [54]	Income from donkey use; impact on social status; empowerment and resilience	Working donkeys in rural and urban Ethiopia contribute to economic security, social status, empowerment and resilience. Donkey income mainly came from pack-carrying and cart-hauling, and their other roles included transport of water and firewood and helping people access areas not accessible by motorised vehicles or that are far away. Income generation was from sale of dung, transport of materials, and agricultural use. Donkey owners contributed to community-based saving schemes which benefited them and other members of the community, and donkeys were highlighted as a pathway out of extreme poverty. Moreover, 80% of donkey owners reported having greater security against environmental and financial hardships. They described their assistance with daily tasks and the ability to gain financial independence and alleviate labour. This was particularly impactful for women.
Geiger et al., 2021 [55]	Participant demographics Use and role of donkey; income from donkeys	Donkey owners used their donkeys primarily for income generating activities in both rural and urban Ethiopia. Rural owners were more likely to own their own house and have a higher number of dependents. Women were the primary users of donkeys in rural locations. Men were the primary users in urban locations. In rural locations, donkeys were mainly used for water carrying, firewood and agricultural purposes. In urban locations, they were mainly used for construction and rubbish collection.
Geiger et al., 2023 [56]	Use of donkeys; economic impact; contribution to participants lives; societal perception of donkeys	Stakeholders described direct and indirect economic impacts of donkeys in rural and urban communities in Ethiopia. The main uses were harvesting and transporting crops, construction, and rubbish collection. Donkey labour enabled people to save money, reduce labour, support family, and contribute to community saving schemes. Participants described personal empowerment through sharing physical labour with donkeys, including the impact on women and children. The impact on donkeys on social status was complex, and depended on the number of donkeys owner, the gender of the owner/carer, and the type of work the donkey was used for.
Geiger 2023 [57]	Participant demographics Economic impact of donkeys; donkeys position in society; social status of donkey owner/co-worker	Women have a key role in donkey care in rural Ethiopia and highly value their contributions to their household and domestic labour. Women described how donkeys supported their families’ income and survival. They had a key role in donkey care and use, but key decisions were often made by men. Donkeys were often referred to as female, even when male, and valued only in terms of labour by men. Both women and donkeys were described as experiencing sexism, subjugation, and violence perpetrated by men, and having lower economic and societal positioning.
Gelaye and Fesseha 2020 [23]	Participant demographics Use of equids	Equids in Central Ethiopia were primarily used for packing and carting and were key sources of income for lower-income or resource-limited respondents. In total, 34% of respondents were illiterate. Equids were used for ceremonial activities. Income was generated from packing (44%), cart transportation (31%), crop threshing (18%), renting (5%), and breeding and selling (2%). The main materials transported were crops and cereals (33%), water (23%), firewood and muck (17%), building materials (13%), charcoal (7%), and vegetables (6%). The average work per week for the equids was 5 days, and the average load was 345 kg for cart horses and 70 kg for pack donkeys. Equids were more important for socially and economically deprived farmers.
Gichure et al., 2020 [8]	Use of donkeys	Donkeys were primarily used for transportation and manure production and contributed to agriculture and trade activities for smallholder farmers in Kenya, important sources of income. Participants described the main benefits of donkeys (in order of highest to lowest ranked) as transport, manure, breeding, ploughing, sale, trading, rent, family asset, identity, and pet. The most frequently transported items were rice and water.
Gichure et al., 2020 [58]	Income from donkeys	In the studied population, 93% of smallholder farmers in central Kenya relied on donkeys as their primary source of income. The daily income from working donkeys was estimated as KES 500 (USD 4.87) compared to KES 100 (USD 0.97) from other livestock, such as cattle, sheep, goats, and chickens. All donkeys were used for transportation of goods by pulling a cart, with additional income from sale of manure. They were considered to have a low cost of maintenance compared to other livestock. Profitability of working donkeys (accounting for costs, including management, treatment, equipment and donkey rearing) was estimated as KES 9270 (USD 90.35) monthly gross margin (KES 300 per day) (USD 2.93), which was 62% of the gross income from working donkeys.
Gina and Tadesse 2015 [59]	Income generation from donkey use; reduction in labour; social status; performance compared to other working animals	Donkeys were the most commonly owned working animals (43%). Other animals owned were cattle and camels; 30% of owners in Ethiopia used working animals to generate income; 53% were used for draught power. Donkeys were considered to have an advantage over other working animals, as they could survive and perform better under drought conditions and when there was limited feed available. Draught power form working animals reduced labour and time taken for women and children performing domestic tasks. Owning a working animal could increase social standing within the community.
Gupta et al., 2017 [60]	Participant demographics Loads carried by donkeys; income generated by donkeys	Donkeys used for brick kiln work in Bihar, India, carry large loads of bricks and generate income for the poorest section of society within these communities. Donkeys carried 25–30 bricks per load, covering 100–500 m per load, and 4–20 km per day. In Patner, during brick-kiln season, each donkey carried 1000–1500 bricks per day, at INR 350/thousand bricks (USD 5.24 ^†^). Each owner earnt about INR 450/day/donkey (USD 6.74 ^†^) and spent about 75/animal/day (USD 1.12 ^†^), but there were seasonal and regional variations.
Gursoy 2020 [61]	Income generation	This qualitative study of horse-drawn carriages described their importance in tourism and for the livelihoods of the coachmen in Izmir and Buyukada in Turkey.
Kithuka et al., 2025 [62]	Income generation	Donkeys contributed 22% to owner incomes on average, the second biggest income source after owning a small business in Kenya. This was an average income of USD 979/household/year from donkeys; 78% owners reportedly had good livelihood incomes.
Koko and Shuiep 2016 [42]	Participant demographics Income generated from donkey use	Most participants from Sudan had no education (52%), or a primary school level (40%). The most common job was as a porter (48%). It was common for children to work with donkeys. 76% of donkeys were used either solely for cart pulling, or cart pulling and farming. Donkeys were used to generate income to support households. The average daily income was SDG 135 (USD 22.17) (range SDG 75–250 (USD 12.32–41.05). The monthly income generated by a single donkey ranged from SDG 2250 to 7500 (USD 369.46–1231.53), with a mean of SDG 4048 (USD 664.70).
Kubasiewicz et al., 2022 [5]	Participant demographics Income from donkeys; financial dependence on equids; income poverty	Donkeys are a key source of income during brick kiln season for owners and thekedars (supervisors) in India. Some participants described donkeys as providing them with a route out of poverty or debt. Most (63%) owners had no formal education. Most owners (97%) and all supervisors relied on donkeys for their main source of income during the kiln season. Donkey owners earnt less than supervisors and more than non-owners during kiln season, and less than both off season. Donkey ownership had provided a route of debt or poverty for some owners, but others struggled due to loss of income and the cost of donkey care out of season. There was a correlation between donkey welfare and income, with lower behavioural scores for donkeys owned by people with lower incomes.
Kubasiewicz et al., 2023 [63]	Owner living conditions Income generation and bonded labour	Donkey owners working in brick kilns in India experience debt-bondage and are highly vulnerable to financial loss. Thekedars (supervisors) earnt INR 187 (USD 2.67) and donkey owners earnt INR 166 (USD 2.37) per 1100 transported bricks and INR 357 (USD 5.10) and INR 327 (USD 4.67) per day, respectively, during brick kiln season. Most owners received advance payments to cover expenses off-season. They described situations of bonded labour, where they were reliant on supervisors for job security, being trapped in cycles of debt, and being exploited or underpaid. Workers described having to migrate to find work during the brick kiln season, and some had children working within the kilns or attending alternative schools during kiln season.
Kubasiewicz et al., 2024 [64]	Income generation Meeting community needs Reliance on animal labour Risks of working with pack mules Potential to meet Sustainable Development Goals (SDGs) [92]	Mules in Nepal have an important role in supporting livelihoods in this mountainous region, as they provide transport to areas that are not accessible by motorised vehicles. Local business owners relied on ‘mule trains’ to deliver stock and supplies, and locals ordered food and necessities. They also transported construction and school materials. Before mules were brought to the region, people had to carry goods and supplies themselves or pay porters, which were expensive. Many mule owners felt working with mules was the only option to be able to provide for their families, as other job opportunities were limited. The majority of mule owners were satisfied with their work, reporting they earnt more than in their previous professions. Several former porters now worked with mules, and working with mules was considered easier than being a porter. Some participants reported that working with mules in these mountainous regions was dangerous for both mules and humans, due to the risk of landslides, unstable tracks, and falling. Few mule owners worked during monsoon season due to increased risks, but this could increase financial struggles during this period. Some mule owners worked during the monsoon season due to necessity or the chance for higher pay, as transport prices nearly doubled. Mules in this region were considered to meet six of the SDGs: no poverty; zero hunger; quality education; decent work; industry, innovation and infrastructure; sustainable cities and communities.
Maggs et al., 2021 [6]	Participant demographics Contribution to livelihood	Donkeys are important for women and children in Ghana contributing to income and saving them time and labour. The main role of donkeys were ploughing (Fielmon communities) and transportation (Gia communities), including water, construction materials, wood, and produce. Income benefits from donkey ownership were described as direct income from hiring out donkey, indirect income from not having to hire for transport of ploughing, collecting and selling firewood, selling donkey products, and saving time through using a donkey for labour. Children play a role in this income generation by accompanying the donkey when it is hired out. Donkeys contributed up to 60% of the household income and saved users up to 6 h a day in time.
Maggs et al., 2023 [65]	Participant demographics Use of donkeys	Donkeys are an important source of income for rural communities in northern Ghana, particularly for women. There are an estimated 14,910 donkeys in Ghana, mainly in northern regions. Their main use was in agriculture (mainly ploughing) and transporting goods (predominantly food items, firewood and water). The majority of respondents said they could not manage without a donkey, and the impact on women and children was particularly noted. Donkey slaughtering for meat and hide was reported to be increasing, with one butcher slaughtering an estimated 1040–1560 donkeys per year.
Merridale-Punter et al., 2024 [66]	Participant demographics Role of equid	This study of cart drivers in Ethiopia described using their working equids for taxi work (median 60%, IQR 0–80%) or transport of goods (median 25%, IQR 10–75%) and water (median 0%, IQR 0–10%). For most, cart driving was their primary occupation, and they derived 100% of their income from this. Most (68%) considered their economic comfort level as ‘just managing’.
Merridale-Punter et al., 2024 [67]	Participant demographics Social and health values of working equids	Women in Ethiopia described the direct and indirect impact of working equids on their health and household through transportation of goods and generation of income. Most women described their economic situation as difficult. They had a median of 3 children, and the mode level of education was early secondary. They had a median of 2 working equids per household. They described direct human health benefits from assistance with physically demanding work, increased household hygiene from water transportation, and benefits to other livestock through transportation of water and feed. They reported direct benefits to their nutrition and water security not only through transportation by donkeys, but also through income generated by working equids to purchase food. Women described increased access to health services either by transporting people, samples, or medicines by working equids, and also by purchasing care and medicines through income generated by working equids. The benefits to communities by sharing working equids and their use when people where ill or pregnant were highlighted. Their impact on wellbeing through labour and time saving was discussed.
Narayanan 2024 [68]	Income generation Bonded labour Reliance on animal labour	Most equid owners at the kilns in India were bonded labourers, paid low wages in advance and having to work to repay the debt to their employer. Equids (and sometimes camels) performed roles in kilns that cannot be mechanised, e.g., in small kilns with shorter distances between the raw bricks and oven only animals could be used to transport the bricks. In a case example given from one kiln, donkey owners had a quota of moving 25,000 bricks to the oven in a day and had to work 16–20 h/day, meaning their donkeys had to carry these bricks and work the same hours. For each trip to the kiln (250–300 m), donkeys were loaded with 25–28 bricks of 5–6 kg each. Owners earnt a rate of INR 160 (USD 1.96) per 1000 bricks. Each donkey earnt their owner USD 1222–2445 per season (6–8 months). In another case example, workers in the kiln received a rate of INR 800 (USD 9.60) per 1000 bricks carried.
Nguekeng et al., 2022 [69]	Participant demographics Reasons for breeding donkeys	Donkeys in Cameroon were mainly bred for use for transportation. The majority of farmers were male, aged between 40 and 60, married, and Muslim. Most had had at least primary education. The mode selling price of donkeys was XAF 140,000–150,000. Agriculture was the main activity for famers, with donkey breeding being a smaller component. Most (92.6%) of the donkeys were bred for transport and agriculture.
Oduori et al., 2025 [70]	Income generation Education level Social value and relationship with equids Roles of equids Impact of donkey theft	On average, 65% of household income was derived from donkey work and use in Kenya; 65% respondents had a primary level of education. Donkeys were used for both income generation, and alleviating some of the labour from household chores. The women highly valued their donkeys, with some examples of participants describing their donkey as a friend, a co-wife who shared the work, and as more important than her spouse in terms of income generation and livelihood support. Donkey theft was an issue in most of the 5 counties samples, with 97% participants from one county experiencing donkey theft in the previous 12 months. These thefts caused negative emotional, health, economic, and wider livelihood consequences. Some participants described feeling bereaved at the loss of their donkey. Women’s health suffered as without a donkey they had to carry heavy loads of water and other items and make multiple trips rather than one. Some women lost their only or major source of income. Respondents in all but one county (the county with the lowest rate of donkey theft) reported a decrease in income; this was by 14%, 38%, 51%, and 73%. Children’s education could be impacted, as families were less able to pay for school fees and learning materials. Children also participated in increased household labour after the theft of a donkey.
Onono and Kithuka 2020 [71]	Income generation Roles of working equids	In Kenya, donkeys contributed an average of 20% of households’ total income from livestock. Donkeys provided a source of employment and a source of generating money by selling them to pay fees for school and medical care. Some also kept donkeys as a sign of prestige or used them to protect their cattle from theft.
Rink and Crow 2021 [72]	Participant demographics Income generation	Cart horses in Cape Town provided a primary source of income for their owners/drivers. This ethnographic study highlights the poverty and social status of the cart drivers. Cart horses were used for removal of construction rubble and garden waste, second-hand and unwanted goods, and scrap metal, and for transporting groceries and parcels.
Sangioni et al., 2016 [39]	Participant demographics Education level Income Equid contribution to communities Working hours, load and mileage of equids	Horses are essential for urban waste recycling in certain communities in Brazil, contributing to income generation and transportation of people and materials. The study highlights the lack of formal education carried out by cart drivers with 71.5% of incomplete primary education level and 46.3% in the age bracket of 10–30 years. Cart drivers had a low income with 70% with an average monthly income below the minimum wage. The load carried had a range of 150–1000 kg, with 36% above 801 kg which is considerably more than the Federal District Degree limit of 350 kg for this type of animal drawn vehicle. 65.8% travelled over 21 km per day, with 60.2% working 1–5 h per day.
Shah et al., 2019 [73]	Participant demographics Use of donkeys Contribution to communities	Donkeys in Pakistan have a key role in waste removal, generating income for their owners and improving sanitation in the communities. The median number of people in each household was 10, and several members would be involved in waste management; 62% of those directly involved were under 18, and 47% were under 14 years of age. Waste collection was the primary income for 89% of donkey owners. Their median monthly income was PKR 7000 (USD 50) per month. Each cart donkey transported a median of 1000 kg non-recyclable waste and 100 kg of recyclable waste per day, and 3142 kg of recyclable waster per month. Most donkey owners (68.5%) reported they dumped waste at official sites. When other households were asked about their waste collection, 49/50 said this was carried out daily by donkey cart, and 53% said there would be a huge garbage build up if the donkey carts did not come.
Tuaruka and Agbolosu 2019 [74]	Roles of working equids	Providing the household with water was ranked as donkeys’ most important role in Ghana, with 56% primarily using their donkeys for this purpose. Access to potable water was a struggle in some communities, with donkeys being used to cart water from rivers and wells. Moreover, 39% generated income from their donkeys through agricultural activities and transporting produce to markets.
Teixeira et al., 2022 [40]	Participant demographics Education level Income Woking hours Types of cargo	Horse and cart drivers play a role in city cleanliness in Southern Brazil and collecting waste materials. In the studied population, 65% had incomplete primary education, with 36.8% initiating their profession under 12 years of age. 36.8% worked 5–7 h per day, with 65.8% carrying miscellaneous cargo. The ban of carts would lead to social and economic exclusion of this marginalised population, with 57.9% receiving their income for their family solely from cart activities. Participants earnt BRL 50–350/fortnight (USD 10.48–73.38 ^†^), with 47.3% receiving less than BRL 250/fortnight of income (USD 52.41 ^†^), less than minimum wage, and supporting more than 5 members per family.
Vasanthakumar et al., 2021 [11]	Participant demographics Role of working equids; source of income; contribution of working equids to income, chores and social interactions	Working equids support women by reducing domestic drudgery, generating income, feeding livestock, and saving time in Guatemala. The women had a median age of 37, and their main income was from agriculture. Most (21/33) had received primary school education only, and 8/33 had had no formal education. Equids contributed to food production by transporting firewood, and food and water for other livestock for all participants. They generated income through transporting wood, fodder and crops. This reduced the household chores, domestic drudgery and labour for the women, but they did also have additional work caring for the equids. Owning and handling equids impacted their standing in the local community, with 17/30 saying it made them more respected.
Wani et al., 2021 [75]	Participant demographics Role of ponies; income generation; contribution to livelihood	Ponies were primarily used for recreational purposes for tourists and livestock rearing, and were a key source of income for ponywallas (horse owners) in Kashmir, India. The majority of ponywallas were illiterate and had received no formal education. They had marginal holdings and most had 5–8 family members. The primary use for the ponies was recreation for tourists (72% in Pahalgam and 58% in Sonamargh), and livestock rearing (8% and 28% respectively). The majority of ponywallas had an income between 11,000 and 20,000 INR/month (USD 146.90–267.09 ^†^), but the amount contributed by ponies was not specified.
Watson et al., 2020 [76]	Participant demographics Impact of donkeys on income and social status	Donkeys were used in brick kilns in Northern India by marginalised communities of low status and poverty. Donkey owners were predominantly male (86%) and aged between 31 and 50 (49%). Brick kiln work was the primary source of income for 97% of participants. Most felt that donkeys increased their perceived status. The brick kiln work resulted in many families travelling long distances, leaving their homes and communities and being vulnerable to poverty and exploitation.
Watson et al., 2022 [77]	Use of mules	Pack mules in Nepal are used for negotiating difficult terrain that cannot be accessed by motorised vehicles and challenging environmental conditions. Before mules began to be used in the region, human power alone was used to transport goods. Mules were purchased from brick kilns, transported and trained to carry packs in mountainous terrain. Working during monsoon season is dangerous for mules and owners, but owners reported needing to continue working or they would not have any earnings to feed themselves and their mules.
Watson et al., 2023 [78]	Participant demographics Use of mules	Mules are used to distribute supplies on the mountain trails in the Gorkha region of Nepal. Most mule owners were male (79%) and aged 30–60 years (67%). Most (83%) mule owners reported that their primary job was working with their mule. The primary role of the mules was pack carrying of goods for households and businesses along the trails, including rice and oil, or construction materials. Mules transported loads too heavy for humans to carry. Owners reported being stressed about the cost of replacing a mule when it died.
Wild et al., 2021 [21]	Participant demographics Income from working equids and impact of Covid-19	Participants across 14 countries reported using working equids across a range of roles and a decrease in income associated with COVID-19. The most common uses of working equids were freight transport, crop transport and transport of people. Equid workload, monthly income from working equids and monthly household income had decreased compared to pre-pandemic. As a result, many had to supplement their income in other ways, such as through extra jobs or money lenders.
**Reports**		
Valette 2014 [13]	Use of working equids Participant rankings of livestock	Working equids were found to be very valuable to women, reducing their labour when doing work and chores, helping them care for their children, providing direct and indirect income generation, and increasing health and social status. Women in 77% of the focus groups across the four countries ranked working equids as their most important livestock, with all groups in India and Kenya ranking them first. The main reason given was due to the regular income they generated. Working equids were also ranked first in 91% groups for their role in helping with household chores. Working equids were described by women as a lifeline and essential to their livelihoods, fulfilling roles both in their household and wider community.
Valette 2015 [79]	Use of working equids Income generated from working equids	Kenya—Food availability was largely determined by donkey-related earning activities. Donkey owners who used donkeys themselves generated the most income from their donkey (USD 2272/year). Casual labourers hired by owners to work with donkeys earnt USD 1389/year, and owners that hired labourers earnt USD 640/year. India—Owners were reliant on their equids for direct income, with equid use being their primary form of income. Equid owning households reported an annual income of USD 1711, of which nearly 80% was directly generated from working animals, with 73% being from brick transportation. Pakistan—Working equids’ roles in supporting owners with agriculture and livestock rearing made owners’ income highly dependent on them. Equids supported 100% of the annual income of households relying on crop and milk sales through their draught power. They underpinned 60% annual income for owners’ relying on labour, crop and milk sales. Estimated household annual income was USD 2500–9000/year. Some households also generated income directly from equid use, which was their primary income. Income directly derived from equids for these households was USD 2000–3000/year. In comparison, limited amounts were spent on equid care (USD 300–500/year), with feed representing the largest cost.

Most publications that reported participants’ income either gave it in USD or provided a conversion to USD. Where a conversion was not provided, the USD equivalent was calculated using historical exchange rate data from Google Finance [93], using the earliest available data point from the relevant month, and rounding to 2 decimal places. * The exchange rate was taken from the month and year that data collection reportedly ended. ^†^ Where this information was not provided, the exchange rate was taken from the month and year that the study was first submitted to the journal for publication.

#### 4.2.2. Educational Interventions

The key features of the 23 eligible publications are presented in Table 5, split by publication type. Eleven publications were journal articles, and twelve were conference contributions. All conference contributions were from a single conference, the 7th International Colloquium on Working Equids, held by World Horse Welfare in 2014 [37]. All studies were conducted in a single country. Ethiopia and India were the most represented countries, with five studies conducted in each, while there were four studies conducted in both Mexico and Pakistan. Lower-middle-income countries were most represented (12/23). All studies were published in English. The interventions in 14 studies were targeted towards only one species of equid, while two targeted horses, donkeys, and mules. Donkeys were included within the intervention target in most studies (10/23). Six of the conference contributions did not specify the species the intervention was targeted towards, which may indicate that these interventions were intended for any equid species. The equids targeted by the interventions were often used for multiple purposes. Twelve studies (12/23) did not specify equid purpose, suggesting interventions targeted working equids broadly within the communities. The majority of interventions were aimed at educating working equid owners and users and sometimes also included other stakeholders such as farriers and members of the local community, including children. Only three did not include owners/users as specifically targeted groups. One of these was aimed at farriers, while one targeted children only, and one targeted children and adult members of the public. Studies employed a range of methods to evaluate the effectiveness of the interventions, with surveys, focus groups, interviews, and equid health and welfare assessments being common. In total, 6 of the 11 peer-reviewed journal articles were published after 2020.

The key results from the 11 journal articles relating to the educational interventions used and their impact are presented in Table 6. Five of these studies assessed the impact of the intervention on the knowledge, attitudes, and/or practices (KAP) of the target population. While the other six directly assessed the impact on the health and welfare of equids, typically in conjunction with human KAP and behaviour change. The latter typically involved more complex and longer-term interventions. Studies targeting KAP typically aimed to improve knowledge of how to care for equids, such as their basic needs, how to prevent and treat injury and disease, or requirements and limits during work. They also promoted positive attitudes towards equids and recognition that they can feel pain and emotions. Participants were encouraged to apply their new knowledge to improve management and work practices. Where specific aspects of equid health and welfare were targeted by the educational interventions, the most common were wounds (5/11), lameness (5/11), and body condition (3/11). Of the studies directly assessing the impact on equid welfare, three aimed to improve overall welfare, including body condition, wounds, lameness, behaviour, and other signs of illness, two focused on lameness only, and one aimed to reduce the prevalence of Epizootic Lymphangitis. Both Reix et al., 2015 [94] and Whay et al., 2015 [95] describe the same intervention, but the former reports the impact on horses’ lameness scores, while the latter explores owner-reported changes in management and lameness. The interventions took on a diverse range of forms, aiming to provide education and encourage change not only through workshops, training programmes, participatory exercises, and village meetings, but also media such as film, audio, handouts, and theatre. Ten studies reported that the intervention had been successful in achieving either some or all of the intended aims. Seven studies involved a participatory element in either the content or design of the intervention or both. Only Makki et al., 2016 [96] reported that the intervention evaluated had not been successful. This was a training programme developed and delivered by the local administration of agriculture, and the authors suggested that training should be tailored to the needs identified by equid users themselves to have a positive impact [96].

**Table 5:** Characteristics of studies identified in a scoping review investigating the impact of educational interventions aimed at improving working equid welfare and/or owner/user knowledge and attitudes. Country income status according to The World Bank 2024 [34].

Author and Year	Main Study Aims	Human Population	Equid Use	Equid Species and No.	Study Methods	Country and Income Status
**Journal Articles**						
Brown et al., 2023 [97]	To use film ethnography for action-research in communities dependent on working equids, aiming to positively influence owner-equid relationships	Local communities working with Animal Nepal: 97 questionnaire participants (48 analysed), 12 interviews, 97 focus group participants	Brick kilns; mountain pack carrying	Mule No equids included in study	Mixed methods: ethnographic film-making, questionnaire to film audience members, interviews, focus groups	Nepal (Lower-Middle)
Duguma et al., 2021 [98]	To trial a community-based approach to understanding and improving mule welfare, with a particular focus on wounds and Epizootic Lymphangitis	Stakeholders—muleteers (mule drivers), muleteer association members, local regulatory officers, business representatives, health professionals 12 key informant interviews; 5 focus groups Total project participants unclear (data presented per year and workshop, not clear whether overlap/attended multiple)	Cart pulling	Mules—no.s varied throughout project (lowest no. sampled was 394 in 2015; highest was 1436 in 2016)	Participatory project management cycle: annual cross-sectional surveys; key informant interviews; focus groups; stakeholder workshops; training and educational interventions	Ethiopia (Low)
Duguma et al., 2025 [99]	To improve donkey welfare through an intervention integrating education and donkey health care to change behaviour and attitudes towards donkeys and to influence policy	8958 donkey-owning households, also stakeholders including local leaders, school children, harness makers, animal health professionals	Pack carrying (9583); cart pulling (3120)	12,703 donkeys	Participatory intervention study: welfare assessments, wound surveys, focus groups used for evaluation	Ethiopia (Low)
Haddy et al., 2021 [100]	To evaluate long term effects of free veterinary treatment, and two educational interventions (farriery courses and handling workshops)	Local communities in three states who had and had not participated in Donkey Sanctuary NGO initiatives: 266 owners/handlers	Riding; pack carrying; agroforestry; sport; other	130 horses; 121 donkeys; 15 mules	Mixed methods: structured interviews and welfare assessments	Mexico (Upper-Middle)
Haddy et al., 2025 [101]	To evaluate the potential of forum theatre as a tool for inclusive community engagement of both adults and children with positive donkey welfare messaging	42 adults; 120 students (aged 11–23 years)	Pack carrying; other non-specified uses	Donkeys No equids included in study	Forum theatre performance Adults: feedback questionnaires Students: pre- and post-performance questionnaires	Kenya (Lower-Middle)
Makki et al., 2016 [96]	To investigate the effect of extension on husbandry, management, and performance of farm draught horses	80 farmers selected from 10 villages	Agriculture	Horses No. not specified	Mixed methods: structured interviews and observations	Sudan (Low)
Reix et al., 2015 [94]	To stimulate and evaluate improvements in lameness and limb abnormalities through a participatory intervention project	439 owners across 21 communities	Ceremonial; transport of goods and people; other work	862 horses	Participatory intervention study: Lameness assessments to evaluate	India (Lower-Middle)
Stringer et al., 2018 [102]	To evaluate the efficacy of knowledge-transfer interventions (audio programme, village meeting, hand out) on long-term (6 month) knowledge change	516 owners from 32 villages	Not specified	Donkeys No equids included in study	Cluster-randomised controlled trial on knowledge-transfer interventions	Ethiopia (Low)
Tadich et al., 2016 [103]	To investigate the recognition of nine basic needs of donkeys by children aged 8–11 years old	173 children aged 8–11 years	Not specified	Donkeys No equids included in study	Educational intervention, children then drew welfare needs and frequency assessed	Mexico (Upper-Middle)
Whay et al., 2015 [95]	To investigate risk factors for lameness in intervention and control groups	439 owners across 21 communities (Statistical analysis on 131 owners)	Not specified—see Reix et al., 2015 [94] for related data	149 horses	Participatory intervention study: interviews to evaluate	India (Lower-Middle)
Yalew et al., 2023 [104]	To compare health and welfare problems between community intervention and non-intervention areas	400 donkey owners/users	Transport, cart pulling	400 donkeys	Cross-sectional study with control group: community-based interventions, welfare assessments	Ethiopia (Low)
**Conference Papers**						
Demissie and Desalegn 2014 [105]	To use the government extension structures to promote simple and easily adaptable husbandry practices among rural equid owners	574 extension workers, 3148 equid owner change agents, 77,289 equid owner followers	Not specified	100,068 equids (species not specified)	Cross-sectional study: extension system assessed using participatory rural appraisal	Ethiopia (Low)
Gogoi et al., 2014 [106]	To describe the gradual transformation in the tetanus toxoid vaccination programme of Brooke India	Equid owners, no.s not specified	Not specified	>60,000 equids (species not specified)	Cross-sectional study: community led health care intervention	India (Lower-Middle)
Granillo and Reyes 2014 [107]	To improve local farriers’ understanding of the hoof, and improve decision-making for shoeing effectively	4 farriers	Rubbish collection	Horses; donkeys; mules No. not specified	Case study: training of 4 farriers with ongoing assessment of trimming and shoeing skills	Mexico (Upper-Middle)
Hassib 2014 [108]	To evaluate needs of working equids in Egypt, and assess the impact of collaborative interventions with a partner organisation designed to address those needs	159 owners for training, 64 owners for welfare assessment, 50 owners for questionnaire	Not specified	63 donkeys; 1 horse	Case study: intervention and wound assessment scoring	Egypt (Lower-Middle)
Madariaga-Najera and Torres-Sevilla 2014 [109]	To (1) change owners’ perceptions and improve the human-equid relationship, through training on equine behaviour; (2) assess the outcome of these training sessions	Mule owners, no. not specified	Not specified	Mules No. not specified	Community behaviour training sessions, observations of mule behaviour and owner testimonies to assess impact	Mexico (Upper-Middle)
Nawaz et al., 2014 [110]	To improve understanding of mass media channels and help identify the best one for increasing awareness amongst equid owners and wider audiences in the future	193 owners first interviews, 211 owners second interviews	Not specified	Species not specified No equids included in study	Equid welfare messages broadcast via radio; structured interviews pre- and post-intervention	Pakistan (Lower-Middle)
Nawaz et al., 2014 [111]	To measure the impact of an educational intervention for equid owners on their working equids and to inform an exit by Brooke from these communities	50 equid owners	Not specified	Species and no. not specified	Mixed methods: structured interviews and focus groups, direct observations of management practices, physical and welfare assessment of equids—measures made pre- and post-intervention	Pakistan (Lower-Middle)
Parai et al., 2014 [112]	To document holistic and appropriate approaches to improve the welfare of working donkeys by changing their feeding practices	Estimated 100 owners attending street play, survey numbers not reported	Not specified	Donkeys No. not specified—estimated impact on 400 donkeys	Mixed methods: observations, intervention—street play containing educational messages, questionnaire, community discussions	India (Lower-Middle)
Pothipongsathorn and Chunekamrai 2014 [113]	To investigate the effects and sustainability of a holistic community intervention on equine health and welfare	Equid owners; community members; farriers Numbers not specified	Cart pulling; breeding	Horses (ponies) No. not specified	Mixed methods: clinical exam, observations, questionnaire, intervention—educational activities	Thailand (Upper-Middle)
Qureshi and Khan 2014 [114]	To identify the role of community-based animal health workers in providing equine health services in areas where no other services are available	14 animal owners trained as community-based animal health workers; 154 equid owning community members participated in focus groups	Not specified	1400 equids, species not specified	Intervention—training of animal health workers and their action in communities, focus groups	Pakistan (Lower-Middle)
Shah et al., 2014 [115]	To assess a community education initiative designed to improve hoof health of cart donkeys	1 owner trained as farrier, 1 trained as community-based animal health worker No. community members and equid owners not specified	Cart pulling	36 donkeys	Baseline health questionnaire; intervention—owner visits by trained farrier and animal health worker; health examinations	Pakistan (Lower-Middle)
Yadav 2014 [116]	To investigate the effectiveness of owner-level foot care training	113 equid owners	Not specified	257 equids, species not specified	Intervention—owner hoof care training; hoof health assessments	India (Lower-Middle)

**Table 6:** Key findings from studies identified in a scoping review investigating the impact of educational interventions aimed at improving working equid welfare and/or owner/user knowledge and attitudes.

Author and Year	Intervention	Evaluation Measures	Key Findings
**Journal Articles**			
Brown et al., 2023 [97]	Ethnographical film making—production of a film representing the typical life stages of a working equid’s life in Nepal, filmed whilst spending time within participating communities. Participatory element where participants asked what should be included in film. Film screenings then organised with these communities. Action development sessions then held in community to make plans of how to improve welfare. These were recorded as posters and displayed in the community.	Pre- and post-screening Likert questionnaires to audience to assess knowledge of equine care; interviews with mule owners and handlers; focus groups held as action development sessions, where participants identified actions they could perform to improve equid welfare.	There was an overall increase in reported knowledge about equine care after the screening. Those reporting they had ‘a lot’ of knowledge increased from 17% to 29%. Thematic analysis of the qualitative data generated themes of ‘before knowledges’ and ‘after knowledges’ (before and after watching the film). ‘Before knowledges’ were defined by a view of mules as an instrument that had minimum care requirements to survive and work. After watching the film, there was increased concern for mules’ individual experiences.
Duguma et al., 2021 [98]	Participatory project management cycle—stakeholders identified and defined problem together, created and implemented a plan for the control and prevention of Epizootic Lymphangitis (EZL), and then monitored and reviewed this. Focus groups and key informant interviews held to understand local perceptions of EZL. Workshops held to agree on a collective intervention and implementation plan. Intervention: education of mule owners on mule care, and EZL prevention and treatment; training of harness makers to reduce wounds from equipment; training of animal health professionals on EZL treatment. Training was ongoing, with follow-ups throughout the project. Euthanasia for advanced cases was introduced as a disease control method (as opposed to abandonment)—training was given to professionals, and awareness raised among the public on the value of euthanasia.	Pre-intervention baseline survey involving clinical examination to measure welfare, and EZL and wound prevalence in mules. Annual follow-up surveys to monitor progress throughout the project (2010–2017). Trainees were assessed using a 4-level competence framework. Regular progress reports were prepared. Stakeholders took part in consultative review workshops. The Regional Livestock Agency, City Administration, and Regional Bureau of Transport, jointly conducted midterm and final evaluations.	Prevalence of EZL decreased significantly from 24% to 6% during the project. Wound prevalence decreased significantly from 44% to 22%. Owner education resulted in increased adoption of recommended EZL prevention and treatment protocols. In total, 8/12 harness makers completed the training and, over the course of the project, produced 584 improved cart saddles, 430 humane bits, and 893 canvas straps and collars. These were exchanged with poor traditional types. All veterinary clinic staff achieved at least the second highest competence level by the end of the project. Obtaining owner consent to euthanise mules with poor prognosis was a challenge, but 123 mules were euthanised during the project. There was a reduction in abandoned EZL-affected mules in the middle of roads and, as a result, a reduction in mule-associated road traffic accidents.
Duguma et al., 2025 [99]	An integrated community-based programme was implemented by The Donkey Sanctuary in Ethiopia, following a participatory programme cycle over five years. Participatory learning and action analysis, and knowledge, attitudes and practice analysis were carried out with stakeholders to identify donkey welfare needs. Problem and solution tree exercises were carried out with stakeholders to identify ways to address problems. Solutions included training animal health professionals and owners in donkey welfare and training harness makers. Suggestions for addressing policy gaps included animal welfare education in schools, improved equine education in veterinary colleges, improving access to services, and lobbying policy makers. Intervention programme aimed to transform main public vet clinics into model clinics and provide training to all animal health professionals in all clinics in target areas. Health care intervention plans developed with main clinic staff. Community education strategies developed with owners and other stakeholders. Development and testing of prototype packsaddle, training of harness makers, who then trained owners or other harness makers. Animal welfare clubs developed as extracurricular activities in primary schools. Project linked with government sectors of agriculture, education, and transport. Exit strategy developed to embed new practices without need for continuous support.	Donkey welfare assessments routinely used (assessing behaviour, body condition, wounds, lameness, other illnesses). Two structured assessments conducted three years apart, where more detailed cross-sectional wound surveys were also carried out. The number of animal health professionals who completed training and who reached independent and trainer level competency was recorded. The impact of the animal welfare clubs on children’s knowledge of donkey care and welfare, attitudes towards donkeys, and their actions towards donkeys and awareness raising in their communities were measured. The Life Skills approach was used to measure success. An animal welfare committee was established, comprising representatives from the government departments of education, livestock, animal health, and transport, and consultations and visits were carried out to monitor the programme.	The intervention resulted in a significant reduction in donkey wound and lameness scores and significant improvement in body condition score across all regions, according to routine welfare assessments. There was also a reduction in wound prevalence from 49.8% to 16.2% found when comparing the early- and late-stage wound surveys. There was also a significant decrease in variation in wound prevalence between regions. The percentages of animal health professionals who reached independent and trainer level competency in each of the three regions reported on were 83%, 81%, and 77%. Dropouts, job changes, and lack of interest in equine health contributed to failure of some to achieve an independent competence level. Children’s understanding of animal welfare and sentience was increased. Their empathy for animals, communication, problem-solving, and confidence also improved. Donkey club members persuaded owners and millers to stop loading donkeys with freshly milled flour, which can become so hot it can burn donkeys, and persuaded mill owners to provide shade and water to donkeys. Children also persuaded parents to take sick donkeys to the vet and influenced communities to carry out de-worming and vaccination. Children also raised awareness of donkey welfare and challenged negative perceptions in communities through role-playing and songs. Regional education and curriculum experts recommended the inclusion of animal welfare education in the national curriculum. The animal welfare committee found that as the programme advanced, many positive changes were being adopted. Donkey owners increasingly engaged with public vet clinics, vaccination centres, and de-worming facilities. Owners adopted improved practices, including using better packsaddles, offloading at market sites so donkeys were not left all day with heavy loads, communicating positively with their donkeys, and providing food, water, and shelter at markets. This led to a significant reduction in donkey welfare cases and fewer abandoned donkeys.
Haddy et al., 2021 [100]	Communities classed as high-, low-, or no-intervention, depending on welfare initiatives that had been conducted by the Donkey Sanctuary NGO. High-intervention initiatives—educational handling workshops or farriery courses run alongside free veterinary clinics. Low-intervention—free veterinary clinics only. All educational interventions had taken place 2–5 years before data collection (other than one which was 10 years before). Veterinary clinics had been running annually or biannually for at least 8 years.	The Equid Assessment Research and Scoping (EARS) tool was used to assess equid welfare and management practices (including physical and behavioural assessment). Structured interviews of 10 questions were held with owners, including questions on participation in welfare initiatives, the role of their equid, beliefs about equid emotions, and the social transmission of equine welfare knowledge in the community.	Equids in high-intervention communities had significantly higher body condition scores and a significantly lower incidence of skin alterations, than those in low- and no-intervention communities. General health status was higher for equids in high intervention communities, but no significant pairwise differences were found between community types. There was no significant difference in lameness across community types. There was no significant difference in behavioural response to the observer across community types. Owners in high-intervention communities were significantly more likely to believe their equid could feel emotions and pain than those in low-intervention communities. No significant difference between high- and no- or no- and low-intervention communities. Owners in high-intervention communities were significantly more likely to ask for advice on their equid and talk about their equid’s health with others than those in low- or no-intervention communities. Results indicate that overall, there was better welfare and increased social transfer of knowledge in communities that had received educational interventions and free veterinary clinics.
Haddy et al., 2025 [101]	Co-creation of a forum theatre intervention. Focus groups held with donkey owners to explore perceptions of donkeys, welfare issues, barriers, and solutions to improving welfare. Co-creation of a drama piece with a local theatre group. Forum theatre was used to encourage audience participation. The narrative was based on real life experiences of struggles in the community, with a ‘bad ending’. The play was then performed again, with audience members invited to intervene and suggest alternative choices which could lead to a better outcome. The aspects of donkey care and welfare featured in the play were as follows: not overloading or beating donkeys, provision of food, water and rest, seeking veterinary treatment for signs of illness, and protection from impacts of ingesting plastic waste. Three public performances were given. The drama piece was also adapted for children and performed in four secondary schools.	Public performances—Short questionnaires were verbally asked to audience members, featuring Likert and open questions. Questions asked whether respondents had enjoyed the performance, if it had raised their awareness about aspects of donkey management and welfare, and the effectiveness of the intervention type at influencing knowledge and behaviour. School performances—Audience members filled out pre- and post-performance questionnaires asking about attitudes towards donkeys and beliefs about their sentience and pain.	Public performances—The majority (88% or higher for all) strongly agreed the play raised their awareness of donkeys’ welfare needs, how much donkeys should carry, how to keep donkeys healthy, and donkeys’ roles in the community. Open questions revealed that 48% reported they had learnt about donkey care and the importance of not mistreating them, and 24% reported learning about the importance of donkeys to the community. In total, 74% reported that they preferred theatre productions as a method of community messaging. School performances—Participants were significantly more likely to report that they liked donkeys, felt confident in identifying how donkeys were feeling, and believed donkeys felt pain in the post- than the pre-performance questionnaires. No significant differences were found for questions about whether donkeys were important, needed rest, felt emotions, needed to be beaten to work, whether they should be loaded as much as possible, and whether participants could identify when they were unwell. In total, 92% believed theatre was an effective way of changing behaviour towards donkeys. From the open questions, 33% reported about learning about the need to take care of donkeys (most common answer). Many participants reported feeling positive emotions while watching the play (48%), while 23% also reported feelings of empathy towards the donkey.
Makki et al., 2016 [96]	Training and extension programme for draught animal technology developed and run by the Administration of Agriculture, EN-Nhoud locality. The authors report the training programmes are run by staff lacking sufficient knowledge. Training packages focus on labour reduction, timeliness, and harnesses and equipment.	Focus groups were held with farmers to gather their perceptions on the training programmes. Verbal questionnaires were asked to farmers about their equid’s health, feeding, and harnessing and also about plough condition. Direct field measurements were used to record working speed, field capacity, and field efficiency.	Focus groups revealed farmers were not satisfied with training quality or the knowledge of training staff. Farmers also wanted training on husbandry practices and work strategies rather than the provided focus of harnessing and equipment. The training was not often conducted in remote villages, requiring farmers to travel, which some could not afford or were unwilling to pay for. Farmers mostly learn about draught work from their peers and experienced farmers. The authors report the questionnaire and field survey revealed that farmers that had taken part in the training programme were not significantly more likely to perform desirable behaviours intended to improve equid welfare, such as regular harness cleaning, offering water during work, and offering more than one feed type to equids. There was also no difference found in farmers’ field performance between those who had and had not completed the training. Overall, the training and extension programme was found to have little impact on farmers’ management and husbandry of draught horses. The authors suggest training needs to be tailored to the needs identified by farmers themselves.
Reix et al., 2015 [94]	Participatory intervention—Facilitator chosen from each community who attended three training workshops on equine welfare and lameness-related issues, involving participatory exercises. Exercises explored husbandry needs and working practices, identifying actions owners could take to reduce lameness risk. Facilitators repeated the exercises and stimulated discussions in meetings with owners from their community. Meetings were held about every 1–2 months over two years, and owners filled in a chart to monitor their progress. A control group did not receive the intervention.	Lameness assessments were carried out with the intervention and control groups at the beginning of the study (before the intervention began), halfway through, and at the end.	Across both groups, only 4% of lameness assessments indicated no lameness. Lameness scores improved in both intervention and control groups across the study period (a lower score indicates less lameness). The improvement was significantly greater in the intervention group. The average overall lameness score in the intervention group improved from 5.1/10 to 3.1/10. In both groups, overall lameness scores increased with age. There was a significantly greater reduction in muscle atrophy across the study in the intervention group. Horses in the intervention group had a significantly greater improvement in range of movement and reduction in pain during joint flexion.
Stringer et al., 2018 [102]	Three knowledge transfer interventions were developed: an audio programme, a village meeting facilitated by a trained animal health worker, and a diagrammatic handout. Participants either received one of these or were part of the no-intervention control group. Intervention content and design described in Stringer et al. 2011 [117]. Participatory situation analysis identified wounds as an owner perceived concern [118], so this was the focus of the interventions. Ten learning objectives were developed relating to the causes, prevention, and treatment of wounds.	Participants answered pre- and post-intervention (approx. 6 months later) questionnaires assessing their knowledge regarding wound prevention and treatment.	All three interventions resulted in a significant improvement in the overall change score between pre- and post-intervention questionnaires compared to the control. The handout and village meeting interventions resulted in significantly greater improvements in knowledge score than the audio programme. The handout also resulted in a significantly greater increase in knowledge score than the village meeting. Increase in knowledge was lower in older participants. The largest overall improvement was seen for the learning objective to be aware of good and bad treatment for wounds.
Tadich et al., 2016 [103]	Teaching of basic needs of donkeys and animal welfare to children, followed by an assessment of understanding through identifying and drawing basic needs on an illustration of a donkey.	Drawings carried out by children to illustrate their knowledge of the basic needs of donkeys at the end of a theoretical training session. The % of children who included each need was calculated by categorising and tallying the need.	In total, 173 children participated in the study, aged 8–11, at 3 primary schools in Tuliman, Mexico. The categories identified were food (100%), water (100%), grooming (81%), shelter/shade (77%), hoof care (75%), human-animal bond (60%), eye care (54%), veterinary services (38%), and bath (35%); the percentage of children drawing each need is indicated in brackets. The study indicated that educational strategies with children could be beneficial, and specific areas of training could be reinforced, such as the importance of veterinary care.
Whay et al., 2015 [95]	Participatory intervention—facilitator chosen from each community to receive training for 10 days using participatory rural appraisal exercises. Groups of 3 facilitators then carried out the same exercises with horse owners within their communities used to identify welfare needs and lameness risk factors. A monitoring chart was created in each community. Facilitators held meetings every 1–2 months for equine welfare discussions over a 2-year period. A control group did not receive the intervention.	Lameness assessments were carried out with the intervention and control groups at the beginning of the study (before the intervention began), halfway through, and at the end. At the final lameness examination, horse owners were interviewed about changes seen in their horses, their equine management practices, and their wider environment. Interviews involved a card-sorting exercise, where owners ordered animal needs into three categories—positive change, no change, and negative change. These were analysed using both qualitative and quantitative methods.	Owners in the intervention group were significantly more likely to report positive changes in equine care for many aspects of husbandry and work than those in the control, e.g., improved diet, increased water provision, better shoeing, reduced working hours, and also an improvement in owner knowledge and a reduction in lameness. In the card sorting exercise, owners from the intervention group indicated that positive changes happened more frequently than the control group. Changes in lameness and limb outcome were described for owners who reported improved management and/or work practices, and these were compared to those who reported no change or a negative change. Some of the reported improvements were associated with improvement in limbs and reduced lameness, whereas others were associated with negative outcomes, giving an inconsistent picture of potential risk factors. This indicates lameness is complex and multifactorial. Owners in the intervention group were engaged and valued the approaches taken, which helped them identify their own solutions.
Yalew et al., 2023 [104]	Community-based interventions were run by an NGO in communities in three districts. The approaches of the community-based interventions aimed to improve the capacity of the communities to improve donkey welfare through education and training of owners, farriers, and equipment makers and by supporting veterinary services. An equal number of communities were selected as a control group.	Physical examination of donkeys to assess body condition score, presence of wounds and lameness, behaviour, and other signs of illness/diseases. The data were compared between donkeys from communities that had received the interventions, and control communities.	There was a significantly lower prevalence of lameness and wounds in intervention than non-intervention communities. Donkeys in the intervention communities had significantly better body condition scores and were more alert and friendly to human approach. Where donkeys had wounds or signs of lameness, these were significantly more severe in the non-intervention than intervention groups.

## 5. Discussion

### 5.1. Overview

This scoping review has comprehensively mapped the available recently published research on both the socioeconomic impact of working equid ownership and the impact of educational interventions targeting working equid owners and users in LMICs. A diverse range of publications were identified for both topics, featuring varying research methodologies and study designs. This reflects the diverse settings in which working equids perform essential roles globally. Donkeys were the most represented species of equid in both review topics, which aligns with data indicating that donkeys are the most populous equid species on average across LMICs [1]. The physical labour of working equids socioeconomically benefited their owners and communities in wide-ranging ways. Those who used equids were typically reliant on them for their livelihoods and survival, including through income generation and labour reduction. The aims and approaches of educational interventions differed, but most reported success. Multilevel initiatives and those developed through participatory engagement may be more likely to be able to demonstrate a direct impact on equid welfare. Varying terminology was used to describe working equids and the topics of interest. Adoption of standardised terms by authors would increase the discoverability and impact of future publications.

### 5.2. Increasing Research Visibility

The search strategy developed for this review included broad search terms to increase the chances of identifying potentially relevant publications. Diverse language was used to describe working equids. Therefore, it was essential to combine equid-related terms with a variety of work-related terms, identified as being used during trial searching and exploration of the wider literature. Several publications found through citation searching did not contain work-related terms in searchable areas of the publication, while one lacked equid-related terms. This explains why these papers were not identified through the databases and demonstrates the importance of additional search streams for research areas that lack consistent terminology. The lack of universally accepted definitions to describe working equids has been identified as a potential challenge for undertaking and identifying research, policy development, resource allocation, and equine welfare programmes [119]. Raw et al., 2024 [119] investigated the terminology used to describe working equids within peer-reviewed literature, finding significant differences in the terminology used according to the World Bank income classification of the country of focus. Using attributes identified in the literature, Raw et al., 2024 [119] proposed that a working equid should be defined as follows: “any equid engaged in physical labour that provides a significant or direct contribution to the economic livelihood, sustenance or support of the owner/user’s family, typically within a low resource setting”. To increase the discoverability of evidence by researchers, policy makers, and other stakeholders, authors should include the term “working equid” in their title, abstract, or keywords when equids featuring within their publication meet this definition.

Similarly, diverse terminology was used to describe educational interventions and the socioeconomic contributions of working equids. It is also recommended that the terms “socioeconomic”, “economic”, or “social” be included within the title, abstract, or key terms of publications exploring socioeconomic aspects. For those searching for evidence on this topic, the term “livelihood” should be included, as this was commonly used within the included publications. For publishing studies discussing educational interventions, it is recommended to include the term “intervention” and a term related to either “education” or “training” within these same sections. When searching for such studies, it is recommended to also include the term “initiative”, which was also commonly used in publications included in this review. In addition, the country of focus should be included within the title, abstract or key terms. Including standardised terms will increase the chance of the publication being found by interested parties.

A sizeable proportion of the publications included in both review topics were conference contributions, with the vast majority originating from the 7th International Colloquium on Working Equids [37]. Conference contributions were included and had their key study characteristics charted to provide a more comprehensive view when mapping the available literature for both topics [120]. However, these sources often did not include full details of methods and findings, so they did not have their key findings charted as was carried out for studies published in full as scientific papers or reports. The conference contributions identified demonstrate the wide variety of work being undertaken across the world, with the involvement and support of many groups and organisations. Yet only a small proportion of this work seems to be published. Over 10 years later, most of these conference contributions have not been published in full. Where relevant research is conducted but not published in full or at all, it cannot contribute to the available scientific evidence base, limiting its wider impact and the ability for others to benefit from the knowledge generated. The 7th International Colloquium on Working Equids appears to be the last one held. It would be valuable for events such as this to be held in the future, as a wide range of research was showcased, even if not in full, that would otherwise not have been discovered. In addition, similar events in the future should aim to support contributing authors to report their research in full, either as a peer-reviewed publication or as a detailed report if resources are not available for the former.

### 5.3. Socioeconomic Value

The socioeconomic impacts reported in the identified studies included both direct and indirect contributions of working equids. The majority of working equids had essential direct socioeconomic roles, such as transport of firewood and water, crops, and building materials [5,23]. These met fundamental needs for owners and communities and relate directly to the United Nations’ Sustainable Development Goals (UN SDGs) with respect to no poverty, zero hunger, and clean water and sanitation [16]. Other roles had indirect impacts, such as the transport of rubbish, which generated income for the owner and improved sanitation for communities [51], or pulling carriages for tourism, providing a source of income for families [61], which again relate directly to UN SDGs. There were regional differences in the roles described for working equids, with studies documenting their use for brick transport primarily from India [5,68,76], and working equid roles in Africa often focused on agricultural use and the transport of water and firewood [6,8]. The role of the working equids in different countries is also aligned with UN SDG priorities for each country. In Ethiopia, for example, a significant proportion of resources is allocated to SDG2, ‘Zero Hunger’ [121]. The most commonly reported roles for equids in Ethiopia relate to agriculture (e.g., transporting crops, cereals and water and crop threshing), again reinforcing the link between working equids and SDGs. The study conducted by Gichure et al., 2020 [58] reported that 93% of smallholder farmers in central Kenya relied on donkeys as their primary source of income, with the daily income from donkeys reported to be five times that from other livestock. The study conducted by Maggs et al., 2021 [6] in Ghana described that donkeys contributed up to 60% of the household income. These studies highlight how working equids have a major role in agriculture in these regions and are essential to the care and farming of other animals.

Most studies reported how working equids were used and/or their economic value. Other studies investigated the social impacts on owners and communities, with several of these having a specific focus on how this affected women and children. The social benefits described included alleviating physical labour, freeing time for other roles and activities, and also enabling access to health care and education (either directly through transport, or indirectly through income generated from the equid) [57,65,67,70]. These impacts relate to a range of other UN SDGs, including good health and wellbeing, quality education, and gender equality [16]. The study conducted by Kubasiewicz et al., 2024 [64] on mules in Nepal directly related their roles to the UN SDGs and identified contributions to the following SDGs: no poverty; zero hunger; quality education; decent work; industry, innovation, and infrastructure; sustainable cities and communities. Although not directly linked to the UN SDGs by the authors, the findings of many other included studies also provide evidence of working equids’ contributions to the same SDGs as Kubasiewicz et al., 2024 [64] and some additional ones. For example, equids in brick kilns in India contributed to no poverty, good health, quality education, and decent work through income generated through their labour, which supported owners and their families [60,63,76]. Equids used for transport and agriculture in Africa also contributed to the following SDGs: no poverty, zero hunger, good health, gender equality, and decent work [41,53,57,65]. Charting the studies for this scoping review has clearly highlighted the importance and value of working equids internationally. This has documented that working equids make major contributions to at least eight of the UN SDGs across several countries. It is also important to note that these impacts are primarily focused on families and communities with low incomes and/or who live in challenging environments, areas where need is often greatest.

The publications on the socioeconomic value of working equids varied widely in their approach and scope, ranging from an ethnographic study of one cart driver and horse [72] to a survey of over 1500 working equid owners across several continents [21]. The variables measured and the reporting of socioeconomic value differed between research studies. This ranged from frequency percentage data on working equid roles to calculations of financial value and qualitative descriptions of their impact on an owner or community [41,53,61]. These reflect the diverse methods of capturing socioeconomic value, but very few studies used similar variables or measures, resulting in a heterogeneous dataset. There were also very few studies which used the same methods to collect and compare data across multiple countries, with only one journal article and two reports carrying this out [13,21,79]. Due to the diverse data types generated, making direct comparisons of working equid socioeconomic contributions between study areas and countries, as well as combining and collating data across different studies and countries, is currently not possible. This contributed to the rationale for performing a scoping review instead of a systematic review for this study. However, this method limits the ability to generate a higher level of evidence. Triangulation between studies and meta-analysis of data from several studies are important to identify areas of consistent large-scale findings. Systematic reviews and meta-analyses combine data to form the highest levels of evidence and should be the goal for future work [122]. However, this will not be achievable unless there is more homogeneity in how data is captured, analysed, and reported.

### 5.4. Educational Interventions

Several interventions aimed to improve working equid health and welfare in general, but wounds and lameness were the most common issues specifically targeted. This aligns with existing research that has identified these as particularly prevalent welfare problems, affecting working equids in a range of countries and settings [19,20,123,124]. One intervention programme, based in Ethiopia, aimed to reduce the incidence of Epizootic Lymphangitis (EZL) [99]. This also aligns with current research, as EZL is highly prevalent in Ethiopia, which has been the focal country of studies demonstrating the disease’s negative impact [18]. The majority of educational interventions targeted working equid owners or users, which was expected, as owners and handlers spend the most time interacting with equids. Therefore, they are likely to have a strong influence over their welfare, be most affected if the equid cannot work due to poor health, and also be most likely to be injured due to unsafe handling/interactions. Some interventions either included or solely focused on children as the target group [99,101,103,104]. Children often have roles caring for equids, and they were found to positively influence the behaviour of their parents and other adults in their communities in relation to donkey welfare after participating in an educational intervention [99]. This suggests that interventions aiming to improve working equid welfare within target communities should consider including children within their design as well as adults. Other studies have also found that children can positively influence the attitudes and behaviours of their parents. For example, one qualitative Maltese study found that many (though not all) children who had received environmental education at school had influenced their parents to adopt more environmentally friendly behaviours, such as recycling, saving water, and turning off unnecessary lights [125].

There were a range of intervention designs with differing aims. Some interventions consisted of a single event, and these were more likely to evaluate the impact on knowledge and attitudes [101,102,103]. However, those that aimed to investigate the impact on equid health and welfare and on human behaviour change tended to be multifaceted and implemented over longer periods of time [94,95,98,99,100,104]. A systematic review assessing interventions aiming to increase pro-environmental behaviour found that single intervention types were typically not very successful at achieving long-term behaviour change and that providing information alone appeared to increase knowledge and change attitudes, but this often did not result in behaviour change [126]. This could be due to a ‘value-action gap’, where people may have positive intentions to change their behaviour but are constrained by barriers such as lack of money, time, or peer support [127,128]. Future interventions should aim to address barriers that could lead to a value-action gap, for example, by consulting members of the target population to identify barriers to behaviour change and potential ideas to overcome these. Interventions should be context-appropriate, using materials, equipment, and treatments that are affordable and readily available to the target population [129]. Additionally, time management and financial strategies could be built into interventions to increase their long-term sustainability, as well as recruiting influential community ambassadors to encourage and support change [130]. The most effective interventions were found to have multiple levels and targets, such as involving education, feedback, improving infrastructure, and enabling strategies [126]. Similarly, a systematic review of interventions aimed at shifting attitudes and behaviour related to gendered stereotypes identified that multi-session educational interventions and those targeting multiple structural levels were more likely to be successful [131]. Due to the varying aims and evaluation measures used by the included studies, it was not possible to provide a direct comparison of which intervention types were most effective. Follow-up studies would be required to investigate whether positive impacts on knowledge, attitudes, and/or practices (KAP) resulting from educational interventions persisted long term and led to improved equine welfare. However, those able to evaluate the impact on human behaviour and equid welfare tended to share the qualities of successful interventions applied in other fields. Additionally, animal and equid welfare non-governmental organisation (NGO) staff have also identified holistic intervention approaches as the most sustainable [129]. This suggests that, where possible, novel educational interventions aiming to change human behaviour to improve working equid welfare should adopt a multifaceted approach as described here.

Some of the identified interventions involved participatory engagement and co-creation with community members and stakeholder groups [94,95,97,98,99,101,102], which Upjohn et al., 2014 [27] previously recommended in this context. NGO staff with experience developing working equid welfare initiatives have also reported that in order to deliver interventions that achieve long-term success, these should be developed with the target community and tailored to the local context [129]. This included ascertaining priority issues identified by the community, adapting approaches in line with cultural norms, and using available resources when providing training, for example, for wound management [129]. Participatory engagement interventions have been shown to be effective at changing behaviour and improving health outcomes in human health care: for example, reducing neonatal and maternal mortality [132]. Additionally, a systematic review found that interventions of this design type increased child vaccination rates in LMICs, with those involving higher levels of embedded community engagement showing the most consistent positive outcomes [133]. All but 1 of the 11 studies reported in full described that the intervention had achieved either some or all of its aims. Regarding the intervention reported to be unsuccessful, working equid users participating in the evaluation study were reportedly dissatisfied with the content of the training, as well as its quality and the knowledge of staff [96]. Engaging with local working equid users to identify their training priorities and course format preferences may have led to increased engagement and outcomes. Meaningful community involvement throughout intervention design, implementation, and evaluation is recommended when developing future initiatives to increase the likelihood of sustained changes in KAP and improved equid welfare.

## 6. Limitations

There is the possibility that some relevant studies may have been missed if they did not contain the combination of search terms used in their title, abstract, keywords, or indexed terms. There may have also been other studies not indexed within the databases searched, which were also not identified through forwards and backwards citation searching. Databases were only searched using English search terms, meaning that publications in other languages that did not have an English translation of the title and/or abstract would not have been discovered. It was beyond the scope of this study to systematically search the grey literature outside of the database searches. Carrying this out could have increased the number of included studies.

The variation in quality and clarity of some studies, particularly within the socioeconomic impact review, increased the difficulty in identifying and charting key findings. Studies where findings were structured and presented less clearly were more often published in LMICs. However, it was important to include and chart these studies’ results as LMICs were the countries of focus in this scoping review and are where people and communities are most reliant on working equids [1,2,3]. It was also considered important to prevent a bias towards including proportionally more publications from high-income countries. Practices and expectations for presenting scientific research may vary between countries and may differ from what this review’s authors are accustomed to. Nevertheless, future authors should take care to describe their study methods in full and report their findings clearly. This includes indicating which results described are their own, and which are comparisons from other research, particularly when the results and discussion are presented together. Reporting guidelines and checklists are freely available for a wide variety of study designs [134], and their use has been found to improve the reporting quality of publications [135,136]. It is recommended that future researchers identify the most appropriate guidelines and follow the structure outlined to present all relevant study information to allow improved interpretation and replication of the research.

## 7. Conclusions and Recommendations

Key data on two important topics relating to working equid use in LMICs have been collated and charted, providing a resource to support researchers, policymakers, and other stakeholders in search of relevant evidence. Working equids have historically been overlooked during policy and funding decisions. This scoping review has brought together the available evidence on the essential roles working equids play in supporting the livelihoods of their owners/users, their families, and wider communities. The identified studies demonstrate equids’ role in the UN SDGs in LMICs, including no poverty, zero hunger, good health and well-being, quality education, gender equality, clean water and sanitation, decent work, industry, innovation and infrastructure, and sustainable cities and communities. Many of the studies also highlighted the contributions of working equids to women’s empowerment. Therefore, the findings of this review should be used as evidence when lobbying governments and other policymaking bodies to include working equids and their owners within resource and funding allocations and the development of health and welfare programmes. Additionally, this scoping review presents the designs and outcomes of recent educational interventions aimed at improving working equid welfare and/or owner/handler KAP. Researchers and other groups aiming to develop novel interventions should consider the features of previous successful initiatives during the design process. Participatory approaches that involve co-creation of the intervention with members of the target community should be prioritised. Interventions that address multiple structural levels and stakeholder groups and that are conducted and evaluated over a longer period may also have the highest chances of long-term success. Due to the broad search strategy applied, this review included some potentially difficult-to-identify studies which do not contain expected key terms or are not indexed on commonly searched databases. Key terms have been suggested that should be included in the title, abstract, and/or keywords of future publications on these topics to aid discoverability and potential impact.

### Future Recommendations

- We recommend that a database is established to summarise the findings of completed working equid research, and current and proposed studies should be registered. This would increase the accessibility of conducted research for relevant stakeholders, especially for those based outside of research and academia. It could also avoid duplication of work, enable collaboration between different groups, and increase the opportunity for recommendations of previous research to be used to enhance the design of future studies. This will enhance the strength and impact of the evidence base.
- Future publications should include the suggested key terms within commonly searched fields to increase their discoverability.
- We recommend the establishment of a database of key health and welfare issues that commonly affect working equids, including preventative measures and treatment options.
- We recommend that easily understandable and accessible advice for designing and presenting research on the topics covered in this review should be developed. This could be used to aid future authors to conduct and publish high-quality research to strengthen the available evidence base.
- The proceedings from international colloquia and conferences focused on or featuring working equids should be published in journals or on sites which are registered with key searchable scientific databases. Such conferences and events should encourage and support authors to publish the details and findings of their studies in full.

## Figures and Tables

**Table 1:** Inclusion and exclusion criteria used for the socioeconomic value literature search.

Criteria	Inclusion	Exclusion
Population	Working equids: horses/ponies (*Equus caballus*) and donkeys (*Equus asinus*) and their hybrids (mules, hinnies) that are used for supporting their owners’ or community’s livelihoods: for example, through income generation, transport, or subsistence support	Domesticated equids kept for any other purposes, e.g., leisure, sport, use in the military or police, solely for farming and production Wild equids and their hybrids (zebras, wild asses, their hybrids, domesticated-wild equid hybrids)
Population	Owners and carers of working equids and professionals (e.g., farriers) working with working equids, including the communities in which they live	Owners/carers of equids kept for other purposes Publications that do not collect data directly from the study population of working equid owners and their communities *
Interest	Publications where the primary focus is on the socioeconomic value or benefit of working equids to their owners and wider community or where this is an additional topic if the primary focus is different A measure of socioeconomic impact must be described in the methods and reported in the results	Publications where the socioeconomic impact of diseases of working equids is the primary focus, rather than the general socioeconomic impact of working equid ownership ^†^
Context	Publications on the socioeconomic value or benefit of working equids used in low- and middle-income countries	Publications involving working equids used in high-income countries
Context	Publications from 2014 onwards	Publications prior to 2014
Study Design	Qualitative; mixed methods; and observational, experimental, and quasi-experimental studies; case series	Narrative reviews, opinion reviews, single case studies (of individual people/animals), scoping reviews, systematic reviews
Publication Type	Peer-reviewed publications, continuing education journals, conference proceedings where a full report is available, textbook chapters, reports, and national guidance	Unable to obtain full text Grey literature and textbooks that do not describe a study that has been conducted Conference papers where full text is not available or is less than 300 words
Language	Full text available in any language included in DeepL [31] that can be translated to English	

* E.g., where only opinions of external organisations, experts, or opinion groups are gathered. ^†^ Publications about the socioeconomic impact of disease can be included if data on the general socioeconomic value of working equids have also been collected and can be retrieved independently.

**Table 2:** Inclusion and exclusion criteria used for the educational interventions literature search.

Criteria	Inclusion	Exclusion
Population	Working equids: horses/ponies (*Equus caballus*) and donkeys (*Equus asinus*) and their hybrids (mules, hinnies) that are used for supporting their owners’ or community’s livelihoods: for example, through income generation, transport, or subsistence support	Domesticated equids kept for any other purposes, e.g., leisure, sport, use in the military or police, solely for farming and production Wild equids and their hybrids (zebras, wild asses, their hybrids, domesticated-wild equid hybrids)
Population	Owners and carers of working equids, and professionals (e.g., farriers) working with working equids, including the communities in which they live	Owners/carers of equids kept for other purposes Publications that do not collect data directly from the study population of working equid owners and their communities *
Interest	Publications which assess the impact of an educational intervention for working equid owners, professionals (e.g., farriers), and communities aimed at changing management practices of working equids, e.g., with the aim of improving equid welfare or reducing owner injury	Publications only describing educational interventions or their development, but not assessing their impact Publications where the intervention is not described or there is insufficient information about the intervention used or method of evaluation
Context	Publications evaluating educational interventions for working equid owners, professionals, and communities in low- and middle-income countries	Publications involving working equids used in high-income countries
Context	Publications from 2014 onwards	Publications prior to 2014
Study Design	Qualitative; mixed methods; observational, experimental, and quasi-experimental studies; case series	Narrative reviews, opinion reviews, single case studies (of individual people/animals), scoping reviews, systematic reviews
Publication Type	Peer-reviewed publications, continuing education journals, conference proceedings where a full report is available, textbook chapters, reports, and national guidance	Unable to obtain full text Grey literature and textbooks that do not describe a study that has been conducted Conference papers where full text is not available or is less than 300 words
Language	Full text available in any language included in DeepL [31] that can be translated to English	

* E.g., where only opinions of external organisations, experts, or opinion groups are gathered.

## Data Availability

No new data were created or analyzed in this study.
